# Improved Binding Affinity and Pharmacokinetics Enable Sustained Degradation of BCL6 *In Vivo*

**DOI:** 10.1021/acs.jmedchem.1c02175

**Published:** 2022-06-02

**Authors:** Rosemary Huckvale, Alice C. Harnden, Kwai-Ming J. Cheung, Olivier A. Pierrat, Rachel Talbot, Gary M. Box, Alan T. Henley, Alexis K. de Haven Brandon, Albert E. Hallsworth, Michael D. Bright, Hafize Aysin Akpinar, Daniel S. J. Miller, Dalia Tarantino, Sharon Gowan, Angela Hayes, Emma A. Gunnell, Alfie Brennan, Owen A. Davis, Louise D. Johnson, Selby de Klerk, Craig McAndrew, Yann-Vaï Le Bihan, Mirco Meniconi, Rosemary Burke, Vladimir Kirkin, Rob L. M. van Montfort, Florence I. Raynaud, Olivia W. Rossanese, Benjamin R. Bellenie, Swen Hoelder

**Affiliations:** † Cancer Research UK Cancer Therapeutics Unit, The Institute of Cancer Research, London SM2 5NG, U.K.; ‡ Division of Structural Biology, The Institute of Cancer Research, London SM2 5NG, U.K.

## Abstract

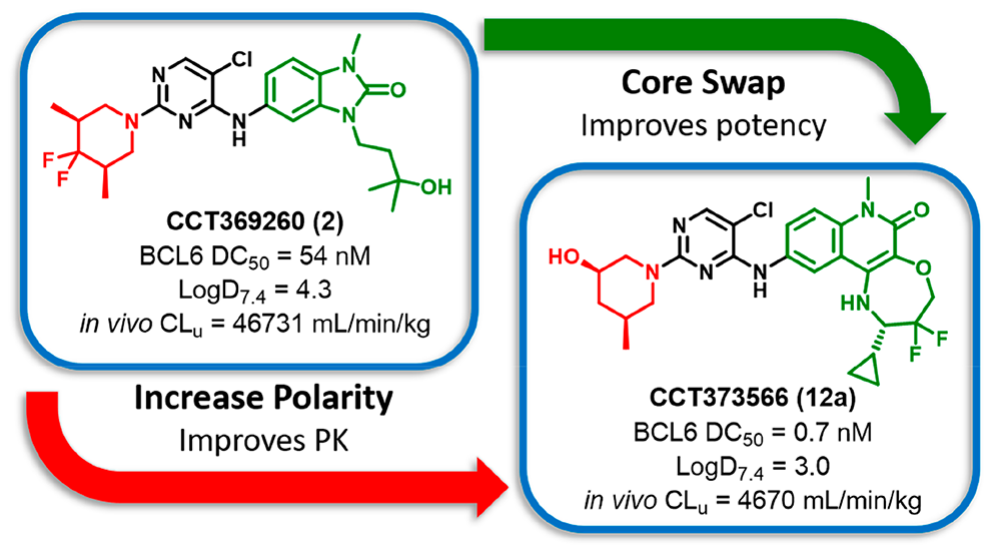

The transcriptional repressor BCL6 is an oncogenic driver found to be deregulated in lymphoid malignancies. Herein, we report the optimization of our previously reported benzimidazolone molecular glue-type degrader **CCT369260** to **CCT373566**, a highly potent probe suitable for sustained depletion of BCL6 *in vivo*. We observed a sharp degradation SAR, where subtle structural changes conveyed the ability to induce degradation of BCL6. **CCT373566** showed modest *in vivo* efficacy in a lymphoma xenograft mouse model following oral dosing.

## Introduction

The formation of germinal centers (GCs) is required for the production of high-affinity antibodies.^1,2^ BCL6 (B-cell lymphoma 6 protein) is a transcriptional repressor highly expressed in GC B-cells. Upon recruitment of one of its corepressors (NCOR, SMRT, or BCOR) to its dimeric BTB domain, BCL6 binds to key sites on DNA and represses genes involved in cell cycle control, cell death, and differentiation.^3,4^ This allows B-cells to proliferate rapidly and evade growth checkpoint controls as required for affinity maturation through somatic hypermutation. Deregulation of these processes through oncogenic BCL6 gene changes leads to B-cell lymphomas, which are dependent on BCL6 expression for survival.^5^ The inhibition of the protein–protein interactions (PPI) between the BTB domain of BCL6 and its corepressors has been targeted to alleviate BCL6-mediated gene repression and discover new treatments for BCL6-driven lymphomas. We and others have pursued inhibitors of the BCL6 BTB domain and demonstrated its ligandability.^6−11^ Importantly, we and others have also reported the serendipitous observation that certain BTB domain inhibitors induce proteasomal degradation of BCL6 in cells. This observation enabled the discovery of small-molecule degraders of BCL6.^12−14^ Furthermore, a recent publication describes that these inhibitors trigger degradation by inducing polymerization of BCL6.^15^

We were intrigued by the possibility of depleting BCL6 from cancer cells and recently reported the degrader **CCT369260**, capable of degrading tumoral BCL6 *in vivo* in lymphoma xenograft mouse models.^14^ However, the high unbound clearance and modest bioavailability of this compound limited its utility in repeated-dose tumor efficacy models. In this study, we report an improved series of BCL6 degraders based on a tricyclic quinolinone scaffold.^11^ This work further elucidated the specific structural requirements and sharp SAR for BCL6 degradation, revealing remarkable stereochemical dependency. Our work ultimately resulted in the discovery of the compound **CCT373566**, a degrader with sub-nanomolar activity and low clearance, which shows strong antiproliferative efficacy in vitro and reduction in tumor growth *in vivo*.

## Results

### Chemistry

**Scheme 1:**
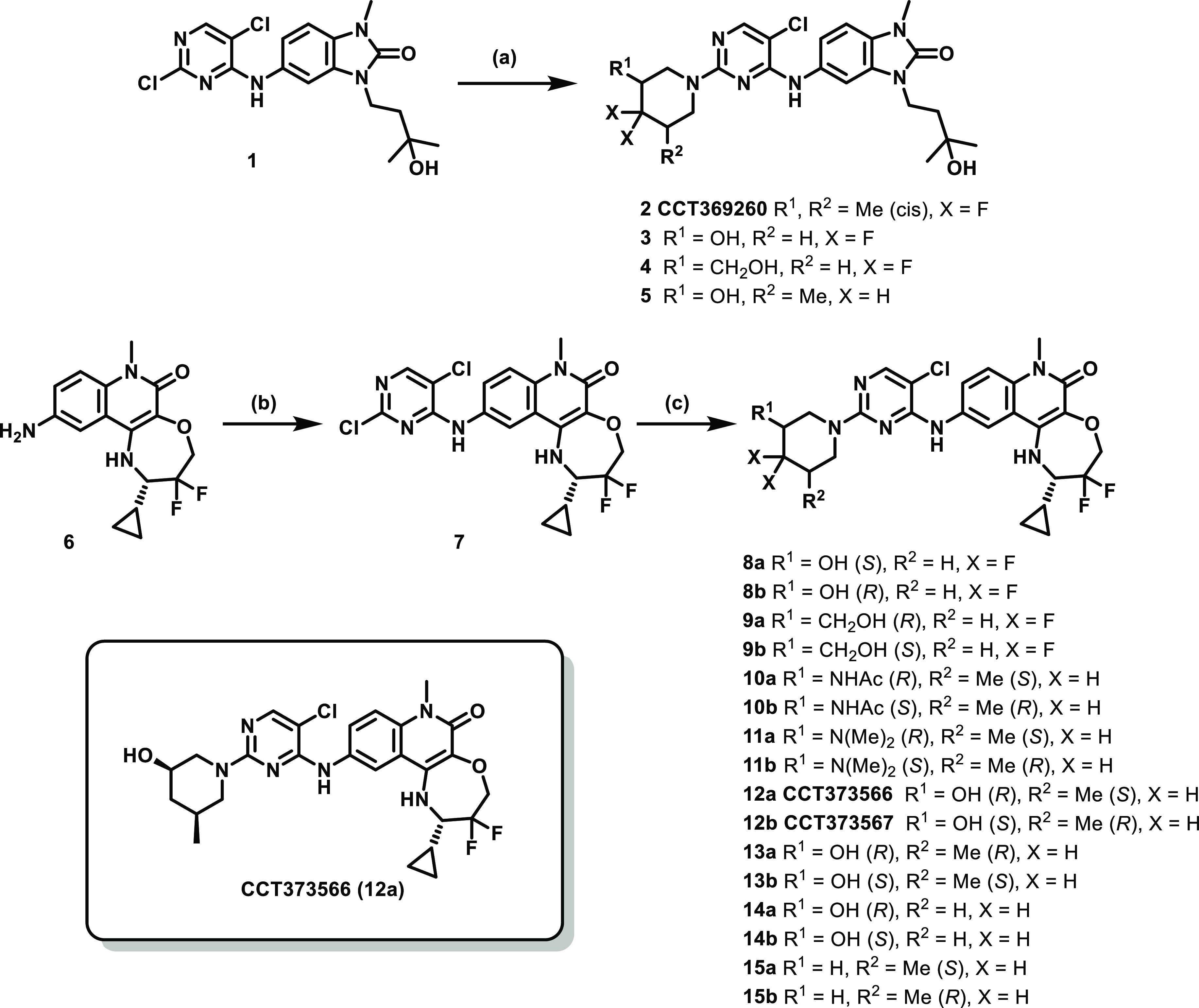
Synthesis of 2-Substituted Pyrimidine Benzimidazolones and Tricyclic Quinolinones from Tables 1–3 Reagents and conditions: (a) cyclic amine, DIPEA, NMP, 140 °C, 1–2 h; (b) 2,4,5-trichloropyrimidine, DIPEA, NMP, 140 °C, 1 h; and (c) cyclic amine, DIPEA, NMP or MeCN, 80–140 °C, 1–18 h.

Final compounds were obtained by a single-step nucleophilic aromatic substitution (S_N_Ar) reaction from dichloropyrimidine intermediates **1** or **7** (Scheme 1). Synthesis of the benzimidazolone intermediate, **1**, has been previously reported.^14^ The tricyclic quinolinone intermediate, **7**, was synthesised *via* a S_N_Ar reaction of trichloropyrimidine with intermediate difluoro aniline **6**.^11^

**Scheme 2:**
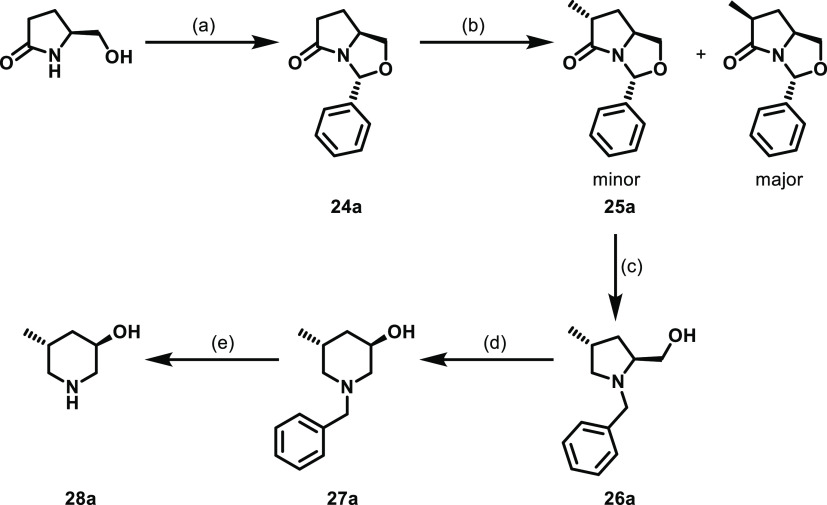
Synthesis of *trans*-5-Methylpiperidin-3-ol (3*R*,5*R*) (**28a**) Reagents and conditions: (a) benzaldehyde, *p*-TsOH, toluene, 120 °C, 16 h; (b) LDA, iodomethane, THF, −78 °C—rt, 4.5 h; (c) LiAlH_4_, THF, rt, 4 h; (d) TFAA, triethylamine, NaOH, THF, −78 °C to 65 °C, 16 h; and (e) Pd/C, H_2_, EtOH, rt, 3 h. The (3*S*,5*S*) isomer was made by a analogous scheme starting from *R*(d)-pyroglutaminol.

Although the *cis*-5-methylpiperidin-3-ol piperidine isomers were commercially available (as a racemic mixture), the *trans*-analogues (**13a**, **13b**) were synthesized from *S*(l)/*R*(d)-pyroglutaminol *via* a previously established route (Scheme 2).^16^ Namely, condensation of *S*(l)-pyroglutaminol with benzaldehyde in the presence of an acid catalyst yields bicyclic intermediate **24a**.^17^ Alkylation with iodomethane results in the formation of both possible isomers, the major cis-isomer and minor desired trans-isomer, **25a**, in a 6:1 ratio, which were readily separated by column chromatography. Reduction of the minor isomer using LiAlH_4_ gave the substituted prolinol, **26a**. Treatment with trifluoroacetic anhydride and triethylamine results in the ring expansion product, **27a**, with the desired stereochemistry retained.^18^ Subsequently, debenzylation by Pd-catalyzed hydrogenation yields the desired *trans*-piperidine required for the final S_N_Ar. This route was undertaken from both *S*- and *R*-pyrogluaminol to give **13a** and **13b**, respectively.

### Reducing Lipophilicity

Our previously leading degrader compound **CCT369260** (**2**) demonstrated depletion of BCL6 in tumors.^14^ However, free plasma concentrations were insufficient to achieve BCL6 degradation 10 h after dosing. Due to the modest solubility of the compound, further increases in the dose did not result in increased exposure. To discover compounds that show more sustained depletion of BCL6 *in vivo*, we set out to reduce clearance and/or significantly improve the potency of degradation (DC_50_) as measured in our cellular BCL6 degradation assay. The log *D*_7.4_ of **CCT369260** was measured to be 4.3 (Table 1), and we hypothesized that reducing lipophilicity may improve PK properties and exposure. A key contributor to the overall lipophilicity of the compound was the 3,5-dimethyl-4,4-difluoro piperidine substituent (calculated to add 2.1 units to the overall log *D*), and we decided to evaluate if less-lipophilic piperidine moieties could be employed. However, we previously found that the substitution pattern of the piperidine ring is critical for the ability to induce degradation.^14^ The challenge to discover less-lipophilic piperidines was thus to introduce polar substituents and remove lipophilic substituents while maintaining potent degradation. Interestingly, in cocrystal structures of piperidine-substituted inhibitors and degraders bound to the BCL6 BTB domain, the piperidine substituent is largely solvent-exposed.^12,14^ The observation that changing the substitution pattern of piperidine strongly affects the degrader SAR is counterintuitive to the observation that no interactions are observed for the piperidine moiety in BCL6 crystal structures. This has led us, and others, to propose that in cells, the piperidine moiety forms additional contacts critical for the formation of ternary or higher order complexes that ultimately drive ubiquitination and degradation. Indeed, Słabicki *et al.* recently proposed that the corresponding 3,5-dimethyl piperidine of the BCL6 degrader BI-3802 can interact with another molecule of the BTB domain homodimer, leading to the formation of higher order BCL6 filaments.^15^ These filaments are then recognized and ubiquitinated by the E3 ligase SIAH1, leading to the proteasomal degradation of BCL6.

**Table 1:**
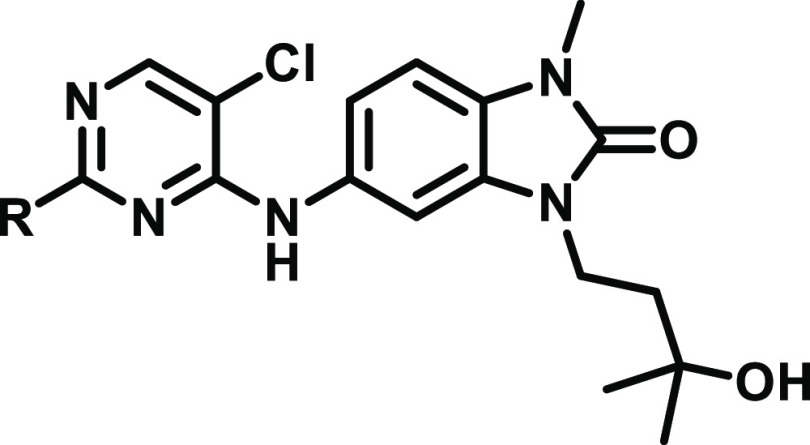

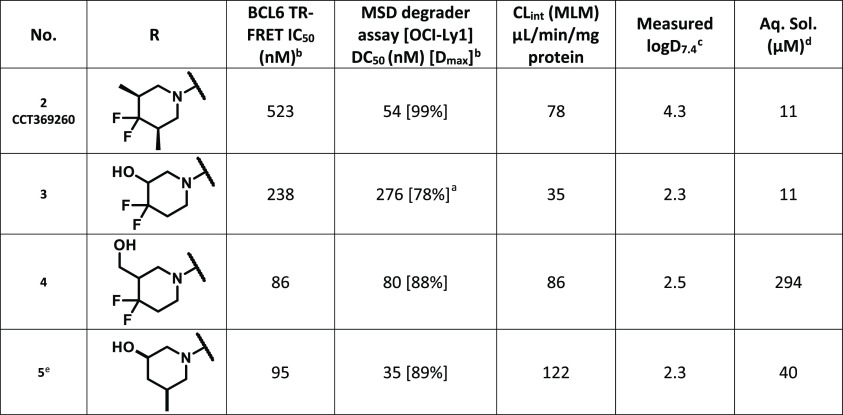
Structure–Activity Relationships of Benzamidazolone Degraders

a Indicates *n* = 2.

b Data represent the geometric mean of at least three replicates. See Tables S1 and S2 for full statistics.

c Measured log *D* determined using the Chrom log *D* method.

d Kinetic solubility measured by NMR in HEPES buffer at pH 8, containing 4% DMSO.

e Compound is a racemic mixture of cis isomers.

Our previous SAR studies had shown that compounds only induced degradation when substituted with a piperidine group that featured a single methyl group in the 5-position or 4,4-difluoro substitution. To obtain less-lipophilic degraders, we thus focused on piperidines that had either of these two substituents and where all other lipophilic substituents were removed or replaced by hydrophilic groups (Table 1). Our goal was to reduce the log *D* by two units while maintaining or improving degradation of BCL6.

The data for the resulting degraders are shown in Table 1. On the whole, our approach to maintain the DC_50_ while reducing lipophilicity succeeded. Particularly, **5** showed a comparable DC_50_ to that of **CCT369260** (35 nM vs 54 nM) but a significantly lower lipophilicity (2.3 vs 4.3). However, this decrease in lipophilicity was generally not accompanied by a reduction in the microsomal clearance. Interestingly, the aqueous solubility was not consistently improved by the reduction in log *D*; no improvement in solubility at all was observed for the hydroxyl difluoro piperidine **3**. In our TR-FRET assay, all three compounds proved to be modestly potent inhibitors of the BCL6 BTB domain. As we observed previously for changes in this part of the molecule, there was no clear correlation between the SAR for degradation and the SAR for biochemical inhibition of peptide binding to the BTB domain. This observation was consistent with the aforementioned role of piperidine in driving the formation of a higher order complex.

With compounds **3**, **4**, and **5**, we had identified significantly less-lipophilic degraders. However, the degradation potency as judged by DC_50_ and the microsomal clearance were still suboptimal. We hypothesized that these properties could be optimized through modification of the benzimidazolone core.

### Optimizing Potency

**Figure 1:**
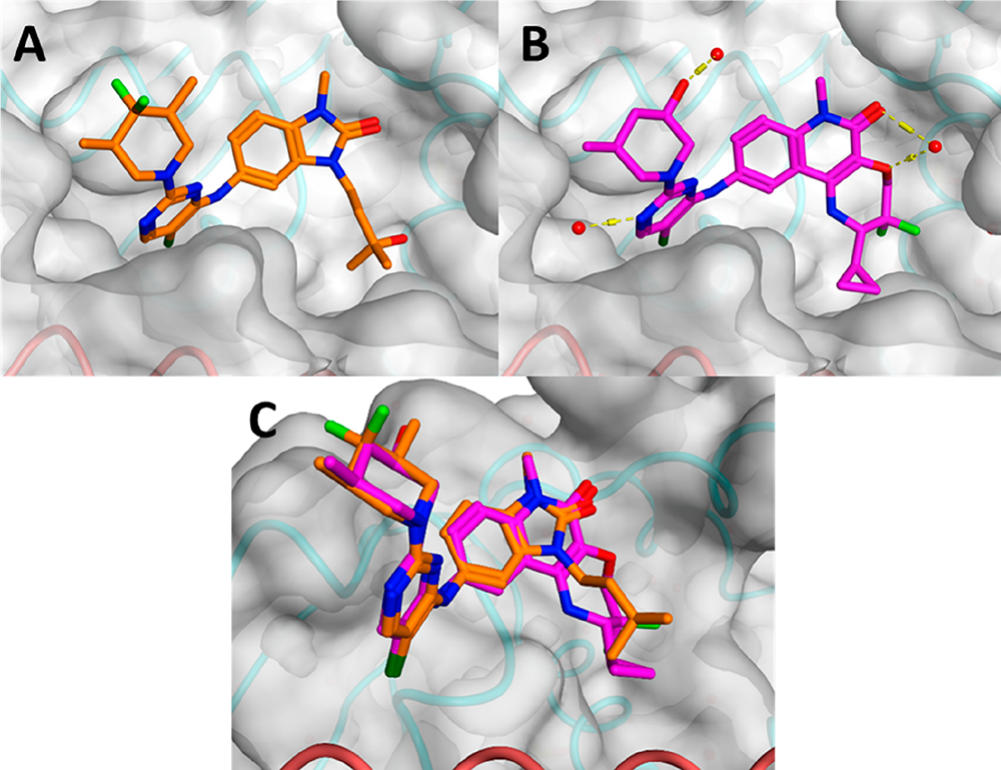
(A) X-ray structure of the BCL6 BTB domain with the bound ligand **CCT369260** (PDB: 6TOM, orange).^14^ (B) X-ray structure of the BCL6 BTB domain with the bound ligand **CCT373566** (PDB: 7QK0, magenta). (C) Overlaid X-ray structures of **CCT369260** and **CCT373566**; the piperidines of both compounds are found to reside in the same position. In all panels, the surface of the BCL6 dimer is shown as a gray transparent surface, with the two individual monomers displayed in ribbons and colored in orange and cyan, respectively. The selected water molecules are shown as red spheres, and H-bonds are shown as yellow dashed lines.

Our crystal structures showed that the benzimidazolone core of **CCT369260** is largely buried in the BTB domain (Figure 1A),^14^ making it unlikely that this part of the molecule engages in interactions with the third component critical for the formation of a higher order complex, for example, with another BCL6 dimer. Because of this observation, we hypothesized that core modifications that result in a tighter inhibitor–protein complex and more potent inhibition of the BTB domain interaction with corepressors will also lead to improved DC_50_ as long as these modifications do not shift the position of the piperidine substituent. To test this hypothesis, we explored our recently discovered tricyclic quinolinone core as an alternative to the benzimidazolone scaffold.^10,11^ BCL6 inhibitors with the new core were found to be over 30-fold more potent in blocking the BTB domain PPI with corepressors than over previous benzimidazolone series. We thus next combined this highly potent core with the more polar piperidine moieties described in Table 1. Furthermore, while the benzimidazolones in Table 1 are racemic mixtures, we prepared pairs of single enantiomers to shed light on how the stereochemistry affects the ability to trigger degradation.

While the switch from benzimidazolone (Table 1) to tricyclic quinolinone (Table 2) was found to increase the log *D*, by maintaining polar substituents on the piperidine, the measured log *D* remained below that of compound **CCT369260**.

**Table 2:**
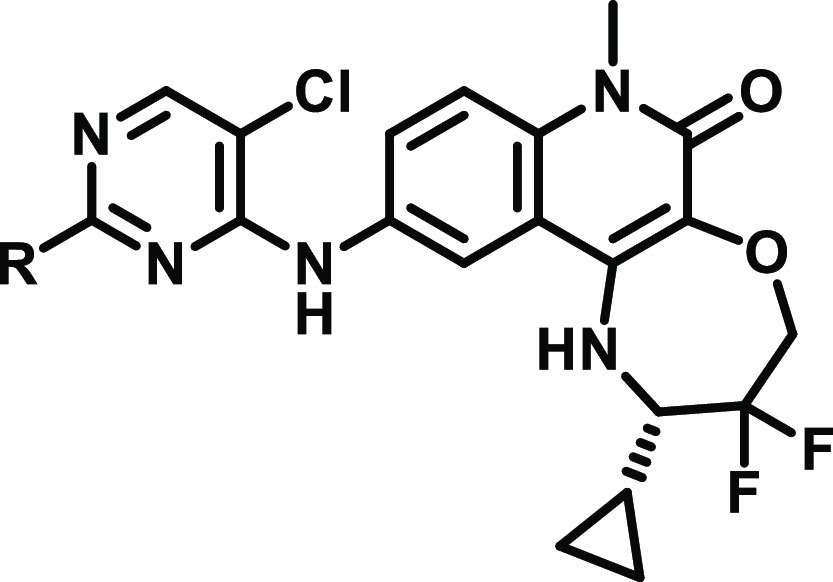

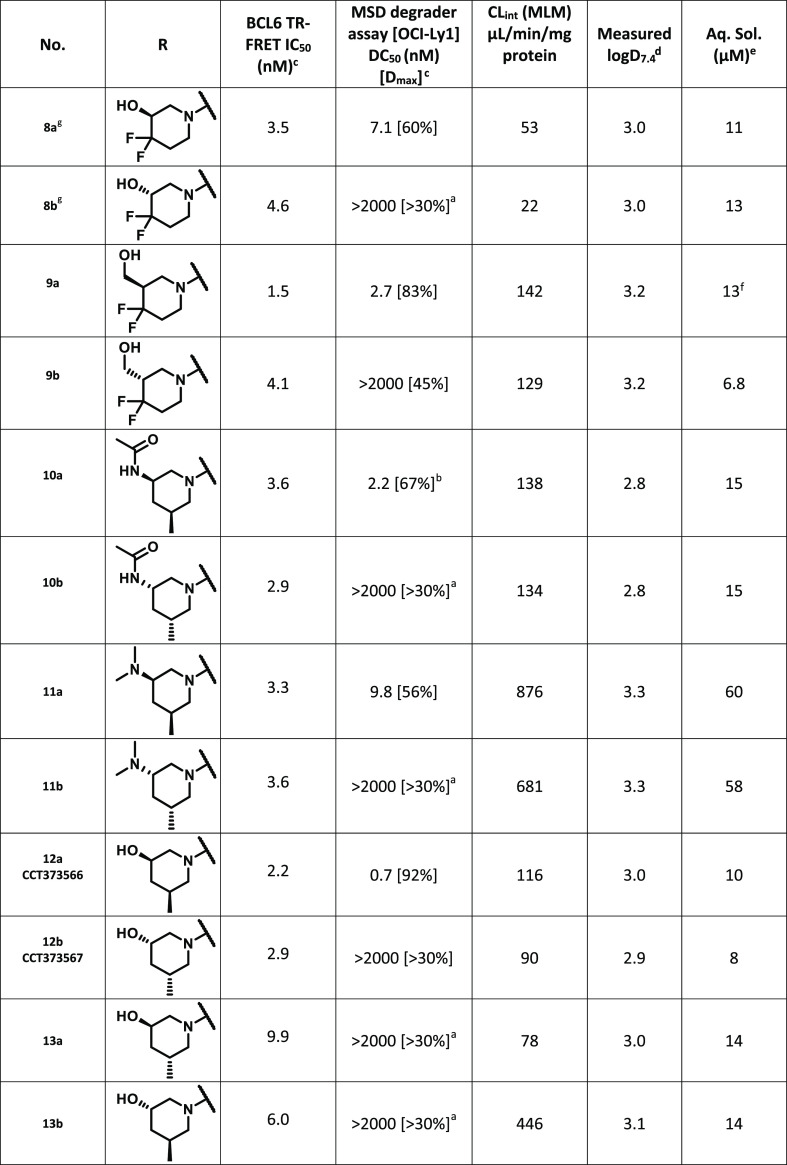
Structure–Activity Relationships of Tricyclic Quinolinone Degraders

a Indicates *n* = 1.

b Indicates *n* = 2.

c Data represent the geometric mean of at least three replicates. See Tables S1 and S2 for full statistics.

d Measured log *D* determined using the Chrom log *D* method.

e Kinetic solubility measured by NMR in HEPES buffer at pH 8, containing 4% DMSO.

f Indicates solubility measured by HPLC in PBS buffer and 1% DMSO at pH 7.4.

g Compound exists as a single unknown enantiomer.

Testing of these compounds (Table 2) revealed clear SAR trends. First, for each of the piperidine substituents, one enantiomer was found to be superior at inducing degradation. This resulted in one nondegrading isomer (**8b**, **10b**, **11b**, and **12b**) and one potently degrading isomer (**8a**, **10a**, **11a**, and **12a**). The difluoro-containing compounds **9a** and **9b** could be considered an exception as both induced degradation of BCL6. However, even in this case, one of the two enantiomers (**9a**) was the superior degrader because it fully degraded the protein, while the opposite enantiomer **9b** only induced partial degradation, with the response plateauing at a maximum of ∼45%.

Second, our assumption that replacing benzimidazolone with the inherently more potent tricyclic core would translate into more potent BCL6 degradation was indeed correct. The core change was found to dramatically increase the potency of inhibition of BCL6, with all compounds demonstrating sub-10 nM activity as measured in the TR-FRET assay. Pleasingly, all active degrading enantiomers showed at least 20-fold lower DC_50_s than those of the corresponding racemic mixture in the benzimidazolone series.

Third, the amide and amine degraders **10a** and **11a** demonstrated that the essential methyl group in the 5-position of the piperidine can be combined with additional polar groups to yield potent degraders. This observation offers the possibility to tweak the overall physicochemical properties of the degraders. However, only incomplete degradation (<90%) of BCL6 was seen for both compound.

Finally, we observed a clear trend for the absolute stereochemistry of the piperidine substituent. As drawn in Table 2, the degrading isomer is the one in which the polar group is “up”, which corresponds to the (*R*) configuration (except for **8a** which is (*S*)). Moreover, piperidines containing methyl groups are only degraders when the methyl is found in the (*S*) configuration.

The most striking SAR was found between the isomers of 3-hydroxy-5-methyl piperidine. The *cis*-3,5-substituted (3*R*,5*S*) piperidine degrader **CCT373566** (**12a**) was found to be our most potent degrader, displaying complete (>90%) and sub-nanomolar degradation of BCL6. In contrast, while showing similar TR-FRET potency, no degradation was seen in the opposite cis-enantiomer (3*S*,5*R*) CCT373567 (**12b**). To further explore this piperidine motif, we targeted the additional two trans-isomers to see if degradation would be lost or maintained (Table 2). While the binding affinity to BCL6 was broadly similar (<10 nM) for each of the possible diastereoisomers, both trans-isomers, **13a** and **13b**, were also found to be nondegraders of BCL6. Only a single isomer of all four possible 3-hydroxy-5-methyl piperidine diastereoisomers, C**CT373566** (3*R*,5*S*-3-hydroxy-5-methyl piperidine), was found to induce any degradation. The remarkable difference between the stereoisomers further defines the essential substitution pattern and pharmacophore that drives degradation of BCL6.

### Probing the Structural Requirements for Degradation

In order to rationalize the SAR observed for substitution of the piperidine moiety, we solved the crystal structure of **CCT373566** bound to BCL6 (Figure 1). Crucially, the resolution of the acquired structure was sufficient to observe the piperidine conformation. As we had established the stereochemistry of the piperidine from the small molecule crystal, this allowed us to distinguish between the similarly sized methyl and hydroxyl groups.

The crystal structure confirmed that the binding of **CCT373566** to BCL6 occurs in the same pocket at the BCL6 BTB dimer interface as seen in our previously published benzimidazolone and quinolinone structures (Figure 1), maintaining the same key interactions (chloropyrimidine clamped between Tyr58 and Asn21, with a π–π interaction with Tyr58, H-bond interaction with Met51, Ala52, and Glu115, and terminal 7-membered ring filling a sub-pocket defined by residues His14, Asp17, Val18, and Cys53 of BCL6) (Figure S1).^10,11,14^ The majority of the molecule (tricyclic quinolinone core) is buried in the BTB domain pocket. The pyrimidine–piperidine moiety aligned well with the corresponding moiety in our previously described degrader **CCT369260** (Figure 1A) with the crucial piperidine substituent largely solvent-exposed. Given that a single methyl group with defined stereochemistry is critical for degradation, we were particularly interested in its position in the **CCT373566** structure. We found that its position was almost identical to the position of one on the methyl substituents of benzimidazolone **CCT369260**, both pointing in the same direction toward the solvent (Figure 1C). No distinct interactions are observed between BCL6 and the piperidine; however, the hydroxyl group does hydrogen-bond to an adjacent solvent water molecule on the surface of the protein. As mentioned above, the lack of interactions of the degrader piperidine with the protein is consistent with the notion that this piperidine forms critical interactions in a higher order complex that is required for degradation but not observed under the soaking conditions employed for crystallography.

To further explore the structural requirements for BCL6 inhibition and degradation, each of the two possible enantiomers of the individual functional groups of the 3-hydroxy 5-methyl piperidine was investigated (Table 3). The hydroxyl piperidines, **14a** and **14b**, inhibited BCL6 with a similar potency to that of **CCT373566**; however, only very partial degradation (35%) was seen for isomer **14a**, and none was seen for **14b**. Interestingly, both monomethyl piperidines, **15a** and **15b**, were shown to be degraders of BCL6 although both the potencies of biochemical inhibition and cellular degradation were significantly decreased compared to those of **CCT373566** (over 10 fold).

**Table 3:**
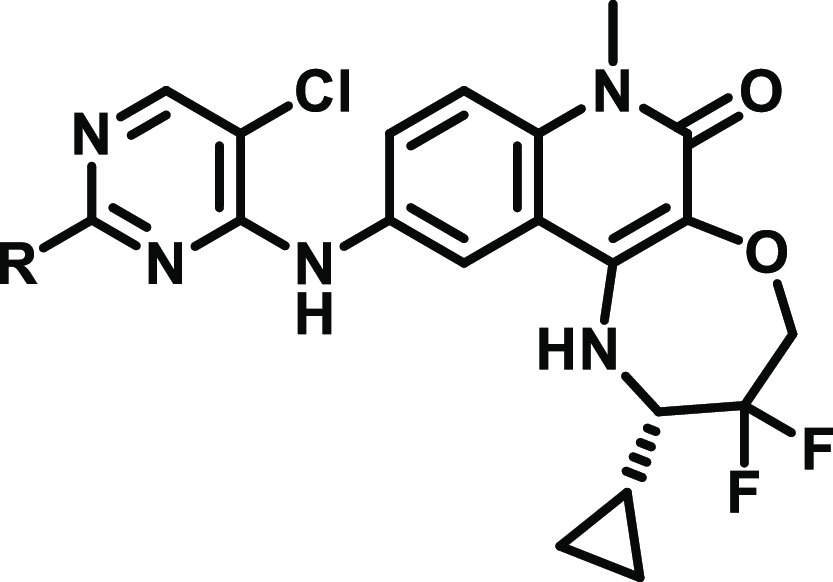

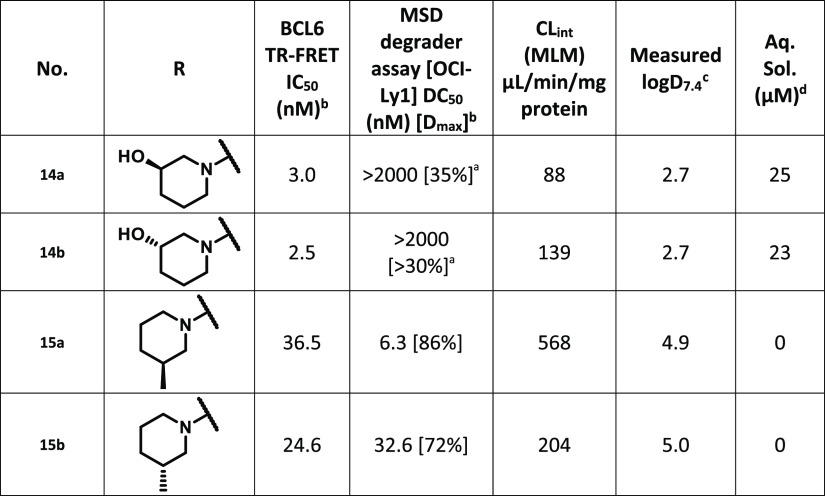
Structure–Activity Relationships of Monosubstituted Piperidine Degraders

a Indicates *n* = 2.

b Data represent the geometric mean of at least three replicates. See Supporting Information Tables S1 and S2 for full statistics.

c Measured log *D* determined using the Chrom log *D* method.

d Kinetic solubility measured by NMR in HEPES buffer at pH 8, containing 4% DMSO.

These results confirmed that the presence of a polar hydroxyl group significantly improves the binding affinity to BCL6 and the methyl group is the key functionality that induces degradation. It is the specific combination of these two groups that leads to the favourable properties of **CCT373566**. The hydroxyl group was shown in the crystal structure to interact with a water molecule on the protein surface. This interaction may explain the increase in potency observed for hydroxyl-containing piperidines, including **14a** and **14b**. Additionally, this interaction may fix the piperidine conformation in place. This could explain why both monomethyl piperidines are still able to induce degradation of BCL6 as both are able to position the crucial methyl group in the right geometry. The conformational locking by the hydroxyl group also helps to rationalize the specificity of the 3-hydroxy 5-methyl piperidine isomers, as it may only be in the case of **CCT373566** that the crucial methyl group is found in the correct position to productively modulate the surface of the BCL6 dimer for degradation.

At this point, we had identified a number of potent degraders in an acceptable lipophilicity range. To assess the potential for further development, we tested microsomal stability in other species and permeation and antiproliferative effects for the selected compounds. The data are summarized in Tables 4 and 5.

**Table 4:** Overview of Key Compounds^a^

	BCL6 TR-FRET IC50 (nM)^b^	MSD degrader assay [OCI-Ly1] DC50 (nM) [*D*max]^b^	Calculated free DC_50_ [OCI-Ly1] (nM)	*f*_u_,media	CL_int_ (HLM/MLM/RLM) μL/min/mg protein	log D7.4^c^	aq. sol. (μM)^d^	PAMPA (pH 7.4)/×10–6 cm s–1
**2 CCT369260**	523	54 [99%]	1.2	0.022	11/78/10	4.3	11	33
**5**	95	35 [89%]	12.4	0.37	28/122/14	2.3	40	23
**9a**	1.5	2.7 [83%]	0.24	0.088	14/142/9	3.2	13^e^	21
**12a CCT373566**	2.2	0.7 [92%]	0.088	0.13	8/116/3	3.0	10	24
**12b CCT373567**	2.9	>2000 [>30%]	NT	NT	3/90/3	2.9	8	16

a NT = not tested.

b Data represent the geometric mean of at least three replicates. See Supporting Information Tables S1 and S2 for full statistics.

c Measured log *D* determined using the Chrom log *D* method.

d Kinetic solubility measured by NMR in HEPES buffer at pH 8, containing 4% DMSO.

e Indicates solubility measured by HPLC in PBS buffer and 1% DMSO at pH 7.4.

**Table 5:** Antiproliferative Activity of Key Compounds

no.	BCL6 TR-FRET IC_50_ (nM)^a^	MSD degrader assay [OCI-Ly1] DC_50_ (nM) [*D*m_ax_]^a^	MSD degrader assay [Karpas 422] DC_50_ (nM) [*D*_max_]	OCI-Ly1 GI_50_ (nM)	Karpas 422 GI_50_ (nM)	HT GI_50_ (nM)	SU-DHL-4 GI_50_ (nM)	OCI-Ly3 GI_50_ (nM)
**12a CCT373566**	2.2	0.7 [92%]	1.0 [85%]	2.1	1.4	8.0	12.5	1900
**12b CCT373567**	2.9	>2000	>2000	83	38	362	820	2145

a Data represent the geometric mean of at least three replicates. See Tables S1 and S2 for full statistics.

In order to compare the potency *in vivo* with the activity observed in our cellular DC_50_ assay, we calculated the free DC_50_ values using the fraction unbound as measured in the OCI-Ly1 DC_50_ assay medium (*f*_u,media_). **CCT373566** was found to be over 13-fold more intrinsically potent than **CCT369260**, which showed higher protein binding.

The mouse microsomal clearance of the tricyclic quinolinone compounds showed no improvement compared to the benzimidazolone series (Tables 1 and 2). However, when measured in other species, the degrader and inhibitor pair, **CCT373566**–**CCT373567**, exhibited low clearance in both human and rat liver microsomes.

The antiproliferative activity of the degrader **CCT373566** and the inhibitor **CCT373567** was tested in a panel of cell lines (Table 5). The degradation of BCL6 by **CCT373566** translated into potent antiproliferative activity. Very potent growth inhibition was observed in 14-day proliferative assays of BCL6 dependent DLBCL cell lines, HT, Karpas 422, SU-DHL-4, and OCI-Ly1 but was not seen in the BCL6 low-expressing cell line OCI-Ly3. **CCT373566** was more potent across all cell lines as compared to the previously reported antiproliferative activity of benzimidazolone **CCT369260**.^14^ While **CCT373567** demonstrated good to modest potency in the highly expressing cell lines, the inhibitor was found to be consistently less potent than its degrading isomer **CCT373566** across the panel of cell lines tested. The increased potency of **CCT373566** compared to that of **CCT373567** indicates that there is an additional advantage to removing BCL6 over inhibition of the recruitment of BCL6 corepressors.

### *In Vivo* Profiling of **CCT373566** (**12a**)

Due to its extremely favorable in vitro degradation and antiproliferative properties, we conducted *in vivo* profiling on **CCT373566**. A pharmacokinetic study, dosing at 1 mg/kg i.v. (*n* = 3) and 5 mg/kg p.o. (*n* = 3), was carried out in female Balb/C mice. All mice appeared normal after dosing and 24 h after dosing. **CCT373566** showed moderate oral bioavailability (44%) and pleasingly low *in vivo* clearance (CL 5.7 mL/min/kg) (Table 6).

**Table 6:** Pharmacokinetic Properties of Compounds **CCT369260** (**2**) and **CCT373566** (**12a**)

	BCL6 TR-FRET IC_50_ (nM)^a^	MSD degrader assay [OCI-Ly1] DC_50_ (nM) [*D*m_ax_]^a^	calculated free DC_50_ [OCI-Ly1](nM)	*C*_max_ (nM/L)	CL (mL/min/kg)	CL_u_ (mL/min/kg)	*t*_1/2_ (h)	*V*_ss_ (L)	*F* (%)	mouse (BALB\c) PPB
**2 CCT369260**	523	54 [100%]	1.2	1577	19.8 (22% Qh)	46731	1.4	2.1	54	99.96
**12a CCT373566**	2.2	0.7 [96%]	0.09	3947	5.7 (6% Qh)	4670	1.15	0.47	44	99.88

a Data represent the geometric mean of at least three replicates. See Tables S1 and S2 for full statistics.

**Figure 2:**
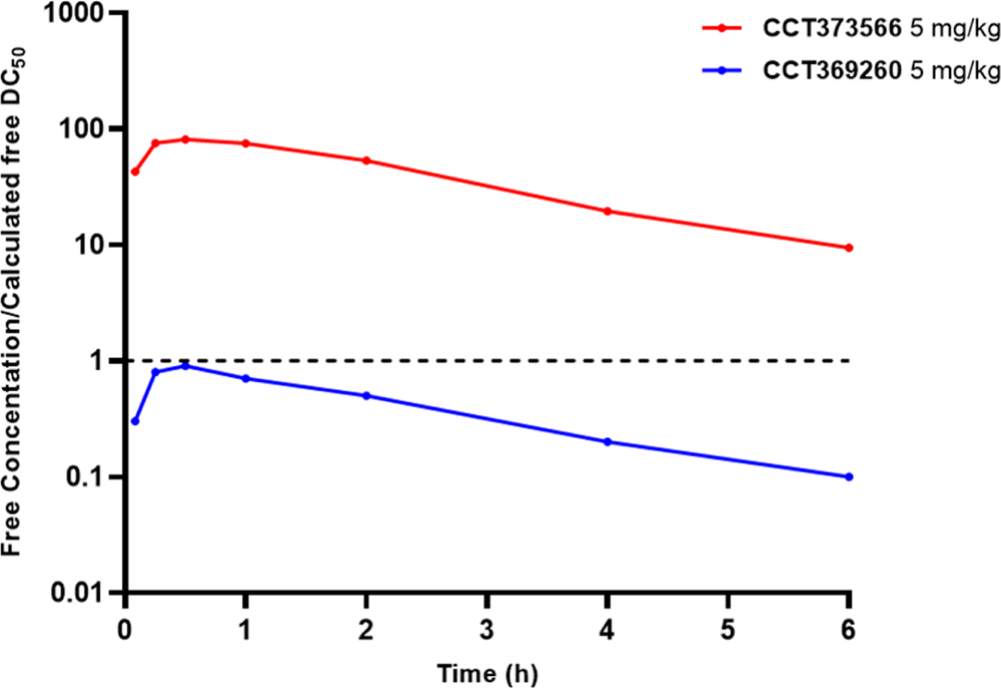
Free mean mouse blood concentrations (nM) divided by the respective calculated free DC_50_ values (nM) of **CCT373566** (red) and **CCT369260** (blue) after po dosing at 5 mg/kg. The dashed, black line represents when a compound is at the free concentration equal to its free DC_50_ value.

When compared to our previously reported degrader, **CCT369260**, the unbound clearance of **CCT373566** was significantly decreased, resulting in an over fivefold increase in free concentration/exposure *in vivo* (Figure S2). Significantly, this decrease in clearance combined with the increase in degradation potency meant that the free concentration of **CCT373566** remained well above the free DC_50_ for 6 h (Figure 2).

A solution formulation was developed to enable the use of higher dosing concentrations. Linearity PK studies in SCID mice demonstrated that exposure increased with the dose and free levels of drug remained above the free DC_50_ for over 16 h when dosed at 50 mg/kg po (Figure S3).

**Figure 3:**
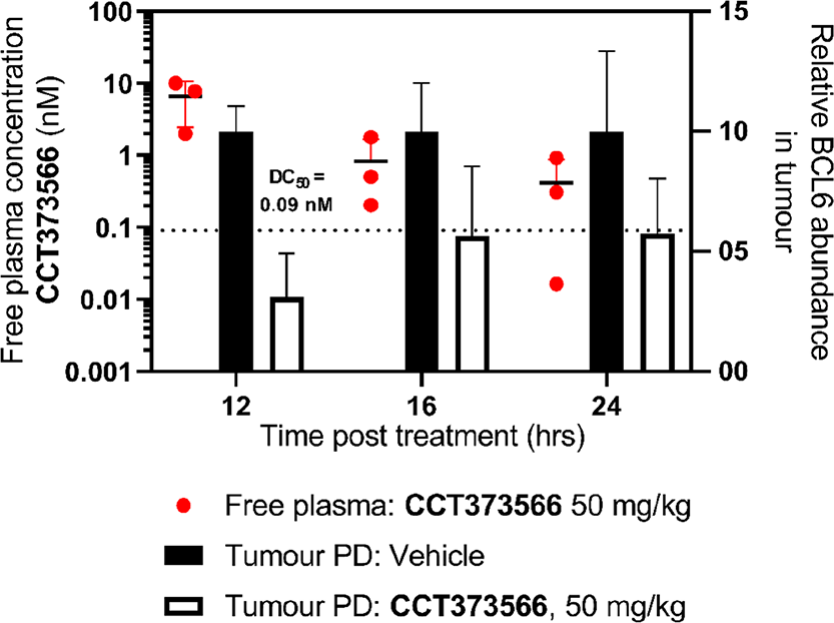
PK/PD study with **CCT373566** at 50 mg/kg po. Tumor xenografts were prepared by subcutaneous injection of 1.5 × 10^7^ OCI-Ly1 cells in female SCID mice, with dosing of the compound commencing 20 days after injection, to mice with xenografts between 0.5 and 0.8 cm^3^, as described in more detail in the Supporting Information. Sampling took place at 12, 16, and 24 h after dosing. All experiments were carried out according to the U.K. guidelines for animal experimentation. BCL6 levels in the tumor were quantified using capillary electrophoresis and normalized to a GAPDH loading control and are shown as black (vehicle-treated) or white (compound-treated) bars. Free compound levels at 12–24 h are shown (red dots); the dotted line indicates free DC_50_.

A PK/PD study in mice using a single dose of **CCT373566** at 50 mg/kg po was undertaken in an OCI-Ly1 DLBCL xenograft model in order to determine the effects on BCL6 levels *in vivo*. No visible adverse events were observed in this study. BCL6 levels were found to be significantly decreased at all time points after dosing (12, 16, and 24 h) with mean free plasma concentrations remaining above the free DC_50_ for 24 h (Figure 3). Tumor concentrations of **CCT373566** were lower than plasma concentrations at all time points consistent with the low *V*_ss_ (0.47 L/kg) seen in the initial PK studies (Figure S4).

**Figure 4:**
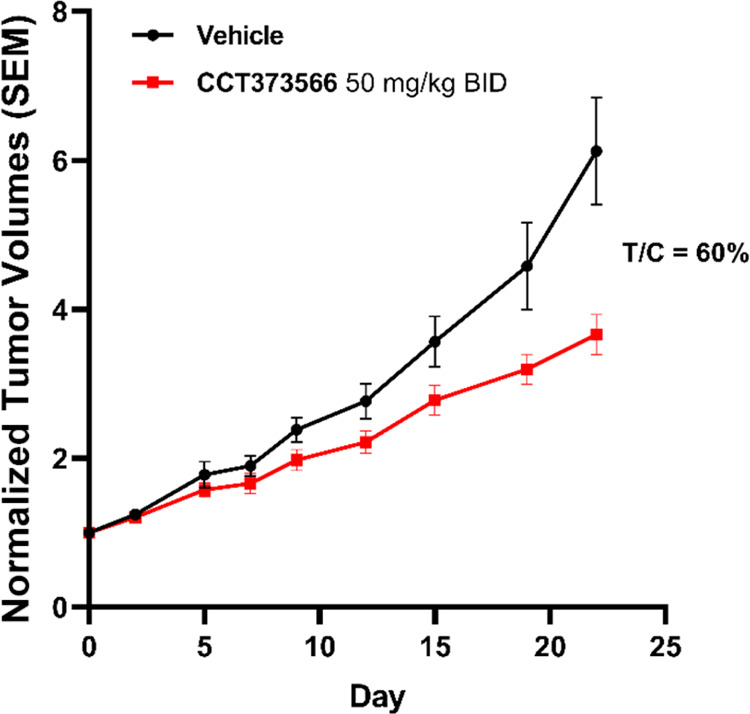
Efficacy study with **CCT373566** at 50 mg/kg po BID for 22 days. Tumor xenografts were prepared by subcutaneous injection of 1 × 10^7^ HT cells in female SCID mice, with dosing of the compound commencing 20 days after injection, to mice with xenografts between 0.5 and 0.8 cm^3^, as described in more detail in the Supporting Information. All experiments were carried out according to the U.K. guidelines for animal experimentation.

Due to the promising *in vivo* PD data, we assessed the antitumor efficacy of **CCT373566** in the HT DLBCL xenograft model in mice. The drug was administered orally (50 mg/kg, bid). No body weight losses were observed in either treatment groups. Post-treatment analysis demonstrated that BCL6 levels remained decreased 4 h and 12 h after the last dose (Figure S5). Somewhat disappointingly, especially given that we had observed sustained depletion of BCL6 and potent antiproliferative effects in vitro, we only observed a modest decrease in tumor growth compared to the vehicle control group (Figure 4). After 22 days of treatment, the tumor growth inhibition ratio (T/C) was 0.6.

Selectivity profiling of **CCT373566** against kinases (468) and a safety panel (78) at 1 μM confirmed selective activity and minimal off-target interactions (see the Supporting Information).

## Conclusions

The goal of our work was to discover a BCL6 degrader capable of sustained depletion of BCL6 *in vivo*. The starting point was our previously disclosed degrader **CCT369260** (**2**). While being a potent degrader, suboptimal PK properties made sustained *in vivo* coverage impossible. With **CCT373566** (**12a**), we ultimately achieved this goal. Two design approaches were key to discovering **CCT373566**: (1) careful, property-focused optimization of the piperidine moiety to reduce lipophilicity while maintaining the ability to degrade BCL6 in cells and (2) replacing the benzimidazolone with our potent tricyclic core that showed significantly tighter binding to the BCL6 BTB domain. Our work further defined the essential substitution pattern of the piperidine moiety needed for potent degradation and, equally important, how polar groups can be incorporated to balance and tweak the overall physicochemical properties. We show that comparatively small changes, for example, changing the stereochemistry of a single methyl substituent, can lead to complete loss of degradation while maintaining potent binding to the BTB domain. The observation that for different piperidine substitution patterns, tightness of binding to the BTB domain does not correlate with degradation potency is in line with the notion that in cells, the piperidine forms additional contacts that are essential for the formation of higher order complexes and that these complexes ultimately drive degradation.

A recent paper shed light on the potential nature of this complex and showed that BCL6 degraders induce polymerization of BCL6 dimers to create higher order filaments. These filaments are then recognized and ubiquitinated by the E3 ligase SIAH1.^15^ Moreover, a model is proposed describing how the piperidine participates in polymerization. The SAR we have observed in our tricyclic quinolinone degraders adds to that previously published and in fact suggests that even more specific structural requirements are needed for degradation.

The crystal structure of **CCT373566** bound to BCL6 showed a binding mode in agreement with our previously reported inhibitors and degraders,^11,14^ with the degradation-inducing piperidine solvent exposed and residing in the same conformation as the piperidine previously seen in the degrader **CCT369260**. One possible rationalization for why degradation is seen in only one isomer of 3-hydroxy 5-methyl piperidine is the hydrogen bond seen between the piperidine hydroxyl group and nearby water that may result in some conformational restriction of the piperidine. The H-bond can only occur in **CCT373566** and **13a** without altering the conformation of the piperidine, and it is only in the case of **CCT373566** that the crucial methyl group is found in the correct position to productively modulate the surface of the BCL6 dimer for degradation.

**CCT373566** emerged as the compound with best overall profile. Notably, it demonstrated much improved *in vivo* clearance and exposure compared to those of **CCT369260**. A PK/PD study showed that 50 mg/kg **CCT373566** dosed orally depleted BCL6 for 12 h and beyond in tumor xenografts. We progressed **CCT373566** to an efficacy study in an HT xenograft model using a twice-daily 50 mg/kg oral dosing scheme. We only observed moderate *in vivo* efficacy in this model. This modest *in vivo* efficacy was surprising given that **CCT373566** showed excellent antiproliferative effects in vitro and BCL6 coverage *in vivo*. However, the result is consistent with a recently published *in vivo* study where BCL6 was depleted by a DOX-inducible knock-out CRISPR/Cas9 system.^19^ In this study also, only modest growth inhibitory effects were observed.

**CCT373566** thus represents an excellent tool to probe the function of BCL6 in human cancer cells and xenografts models. We will publish the efficacy of **CCT373566** in other BCL6-expressing models in due course.

## Experimental Section

All *in vivo* experiments were carried out according to the UK guidelines for animal experimentation. Cell lines were supplied by the German Collection of Microorganisms and Cell Cultures (DSMZ). Cell lines were authenticated by STR profiling and were routinely screened for mycoplasma, using an in-house PCR-based assay.

### General Synthetic Information

All anhydrous solvents and reagents were obtained from commercial suppliers and used without further purification. Evaporation of the solvent was carried out using a rotary evaporator at reduced pressure at a bath temperature of up to 60 °C. Flash column chromatography was carried out using a Biotage purification system using SNAP KP-Sil or Sfar cartridges or in the reverse-phase mode using SNAP Ultra C18 cartridges. Semipreparative separations were carried out using a 1200 Series preparative HPLC over a 15 min gradient elution. Microwave-assisted reactions were carried out using a Biotage Initiator microwave system. The final compounds were purified to ≥95% purity. NMR data were collected on a Bruker Avance 500 spectrometer equipped with a 5 mm BBO/QNP probe or on a Bruker Avance Neo 600 spectrometer equipped with a 5 mm TCI Cryo-Probe. NMR data are presented in the form of chemical shift δ (multiplicity, coupling constants, and integration) for major diagnostic protons, given in parts per million (ppm) relative to tetramethylsilane (TMS), referenced to the internal deuterated solvent. HRMS was assessed using an Agilent 1200 series HPLC and diode array detector coupled to a 6120 time of flight mass spectrometer with a dual multimode APCI/ESI source or on a Waters Acquity UHPLC and diode array detector coupled to a Waters G2 QToF mass spectrometer fitted with a multimode ESI/APCI source.

### Preparation of Compounds

Final compounds containing racemic or meso piperidine substituents (**2** (**CCT369260**), **3**, **4**, and **5**) were synthesized from commercially available building blocks. Single-diastereoisomer compounds were synthesized from commercially available single-enantiomer piperidines (**10a**, **10b**, **11a**, **11b**, **14a**, **14b**, **15a**, and **15b**) and enantiopure, protected piperidines and separated by chiral SFC (**9a**, **9b**, **12a** (**CCT373566**), and **12b** (**CCT373567**)) or were synthesised racemic and separated as diastereoisomers by HPLC (**8a** and **8b**).

#### 5-((5-Chloro-2-((3*R*,5*S*)-4,4-difluoro-3,5-dimethylpiperidin-1-yl)pyrimidin-4-yl)amino)-3-(3-hydroxy-3-methylbutyl)-1-methyl-1,3-dihydro-2*H*-benzo[*d*]imidazol-2-one (**2**, **CCT369260**)

As previously published.^14^

#### 5-((5-Chloro-2-(4,4-difluoro-3-hydroxypiperidin-1-yl)pyrimidin-4-yl)amino)-3-(3-hydroxy-3-methylbutyl)-1-methyl-1,3-dihydro-*2H*-benzo[*d*]imidazol-2-one (**3**)

A mixture of 5-((2,5-dichloropyrimidin-4-yl)amino)-3-(3-hydroxy-3-methylbutyl)-1-methyl-1,3-dihydro-2*H*-benzo[*d*]imidazol-2-one (**1**) (25 mg, 0.063 mmol), 4,4-difluoropiperidin-3-ol hydrochloride (33 mg, 0.19 mmol), and DIPEA (44 μL, 0.25 mmol) in NMP (1.5 mL) was heated under microwave irradiation to 140 °C for 1 h. The resulting mixture was purified using an Agilent 6120 MS-Prep LC (ACE 5 C18-PFP 250 × 21.2 mm column using a 15 min gradient of water/methanol (both modified with 0.1% formic acid) and eluted from 60:40 to 0:100 at a flow rate of 20 mL/min) to give **3** (23 mg, 0.046 mmol, 73%). HRMS (ESI +ve): found 497.1890 expected 497.1874 for C_22_H_28_ClF_2_N_6_O_3_ [M + H]^+^; δ_H_ (600 MHz, CD_3_OD): 7.93 (s, 1H), 7.56–7.47 (m, 1H), 7.32–7.27 (m, 1H), 7.11 (d, *J* = 8.5 Hz, 1H), 4.08–3.94 (m, 3H), 3.90–3.84 (m, 1H), 3.82–3.71 (m, 3H), 3.43 (s, 3H), 2.22–2.10 (m, 1H), 1.97–1.78 (m, 3H), 1.29 (s, 6H).

#### 5-((5-Chloro-2-(4,4-difluoro-3-(hydroxymethyl)piperidin-1-yl)pyrimidin-4-yl)amino)-3-(3-hydroxy-3-methylbutyl)-1-methyl-1,3-dihydro-2*H*-benzo[*d*]imidazol-2-one (**4**)

The method is the same as for **3**, using (4,4-difluoro-3-piperidyl)methanol. Purification by preparative HPLC (ACE 5 C18-PFP 250 × 21.2 mm column; 15 min gradient of 90:10 to 0:100 water/methanol (both modified with 0.1% formic acid) at a flow rate of 20 mL/min) was performed to give **4** (13 mg, 0.025 mmol, 65%) as formic acid salt. HRMS (ESI +ve): found 511.2036 expected 511.2030 for C_23_H_30_ClF_2_N_6_O_3_^+^ [M + H]^+^; δ_H_ (600 MHz, CD_3_OD): 8.22 (s, formate), 7.95 (s, 1H), 7.43 (d, *J* = 1.9 Hz, 1H), 7.41 (dd, *J* = 8.4, 1.9 Hz, 1H), 7.12 (d, *J* = 8.4 Hz, 1H), 4.50 (br d, *J* = 13.6 Hz, 1H), 4.26 (m, 1H), 4.03 (m, 2H), 3.88 (dd, *J* = 11.2, 4.1 Hz, 1H), 3.48 (dd, *J* = 11.2, 9.2 Hz, 1H), 3.44 (m, 1H) overlapping with 3.43 (s, 3H), 3.31 (dd, *J* = 13.6, 9.4 Hz, 1H), 2.13 (m, 1H), 2.01 (m, 1H), 1.96–1.86 (m, 1H) overlapping with 1.87 (m, 2H), 1.30 (s, 6H).

#### 5-((5-Chloro-2-(3-hydroxy-5-methylpiperidin-1-yl)pyrimidin-4-yl)amino)-3-(3-hydroxy-3-methylbutyl)-1-methyl-1,3-dihydro-2*H*-benzo[*d*]imidazol-2-one (**5**)

The method is the same as for **3**, using *rac*-5-methylpiperidin-3-ol. Purification by preparative HPLC (ACE 5 C18-PFP 250 × 21.2 mm column; 15 min gradient of 90:10 to 0:100 water/methanol (both modified with 0.1% formic acid) at a flow rate of 20 mL/min) was performed to give **5** (13 mg, 0.027 mmol, 90%) as the formic acid salt. HRMS (ESI +ve): found 475.2224 expected 475.2219 for C_23_H_32_ClN_6_O_3_^+^ [M + H]^+^; δ_H_ (600 MHz, CD_3_OD): 8.14 (s, formate), 7.90 (s, 1H), 7.51 (d, *J* = 2.0 Hz, 1H), 7.33 (dd, *J* = 8.4, 2.0 Hz, 1H), 7.12 (d, *J* = 8.4 Hz, 1H), 4.73 (ddt, *J* = 12.4, 5.0, 1.8 Hz, 1H), 4.48 (ddt, *J* = 13.0, 3.9, 1.7 Hz, 1H), 4.13–3.97 (m, 2H), 3.54 (tt, *J* = 10.6, 4.6 Hz, 1H), 3.43 (s, 3H), 2.48 (dd, *J* = 12.4, 10.6 Hz, 1H), 2.29 (dd, *J* = 13.0, 11.4 Hz, 1H), 2.11–2.03 (m, 1H), 1.94–1.82 (m, 2H), 1.68–1.57 (m, 1H), 1.29 (s, 6H), 1.07 (q, *J* = 11.4 Hz, 1H), 0.95 (d, *J* = 6.6 Hz, 3H).

#### (*S*)-2-Cyclopropyl-10-((2,5-dichloropyrimidin-4-yl)amino)-3,3-difluoro-7-methyl-1,2,3,4-tetrahydro-[1,4]oxazepino[2,3-*c*]quinolin-6(7*H*)-one (**7**)

A mixture of (2*S*)-10-amino-2-cyclopropyl-3,3-difluoro-7-methyl-2,4-dihydro-1*H*-[1,4]oxazepino[2,3-*c*]quinolin-6-one (**6**)^11^ (1.70 g, 5.29 mmol), 2,4,5-trichloropyrimidine (0.67 mL, 5.82 mmol), and DIPEA (3.7 mL, 21.23 mmol) in NMP (5 mL) was heated under microwave irradiation to 140 °C for 1 h. The reaction mixture was allowed to cool to rt, and water (15 mL) was added. The resulting precipitate was filtered and washed with water (20 mL). The solid was purified by flash column chromatography (Biotage 50 g KP-sil, 0–10% MeOH in DCM) to give **7** as a pale-yellow solid (2.4 g, 5.14 mmol, 97%). HRMS (ESI +ve): found 468.0784 expected 468.0800 for C_20_H_18_Cl_2_N_5_O_2_^+^ [M + H]^+^; δ_H_ (500 MHz, DMSO-*d*_6_): δ 9.74 (s, 1H), 8.38 (s, 1H), 8.15 (d, *J* = 2.3 Hz, 1H), 7.60 (dd, *J* = 8.9, 2.2 Hz, 1H), 7.50 (d, *J* = 9.0 Hz, 1H), 6.31–6.16 (m, 1H), 4.60–4.26 (m, 2H), 3.58 (s, 3H), 3.30–3.19 (m, 1H), 1.38–1.22 (m, 1H), 0.83–0.63 (m, 1H), 0.58–0.45 (m, 2H), 0.39–0.28 (m, 1H).

#### *rac*-(2*S*)-10-((5-Chloro-2-(4,4-difluoro-3-hydroxypiperidin-1-yl)pyrimidin-4-yl)amino)-2-cyclopropyl-3,3-difluoro-7-methyl-1,2,3,4-tetrahydro-[1,4]oxazepino[2,3-*c*]quinolin-6(7*H*)-one (**8**)

A mixture of (*S*)-2-cyclopropyl-10-((2,5-dichloropyrimidin-4-yl)amino)-3,3-difluoro-7-methyl-1,2,3,4-tetrahydro-[1,4]oxazepino[2,3-*c*]quinolin-6(7H)-one (**7**) (25 mg, 0.063 mmol), *rac*-4,4-difluoropiperidin-3-ol hydrochloride (30 mg, 0.18 mmol), and DIPEA (61 μL, 0.35 mmol) in NMP (1.5 mL) was heated under microwave irradiation to 140 °C for 1 h. The resulting mixture was purified by preparative HPLC (ACE 5 C18-PFP 250 × 21.2 mm column; 15 min gradient of 90:10 to 0:100 water/methanol (both modified with 0.1% formic acid) at a flow rate of 20 mL/min) and further by SCX-2 to give **8** (mixture of diastereoisomers) as a beige solid (20 mg, 0.035 mmol, 40%).

#### (*S*)-10-((5-Chloro-2-((*S*)-4,4-difluoro-3-hydroxypiperidin-1-yl)pyrimidin-4-yl)amino)-2-cyclopropyl-3,3-difluoro-7-methyl-1,2,3,4-tetrahydro-[1,4]oxazepino[2,3-*c*]quinolin-6(7*H*)-one (**8a**) and (*S*)-10-((5-Chloro-2-((*R*)-4,4-difluoro-3-hydroxypiperidin-1-yl)pyrimidin-4-yl)amino)-2-cyclopropyl-3,3-difluoro-7-methyl-1,2,3,4-tetrahydro-[1,4]oxazepino[2,3-*c*]quinolin-6(7*H*)-one (**8b**)

*rac*-(2*S*)-10-((5-Chloro-2-(4,4-difluoro-3-hydroxypiperidin-1-yl)pyrimidin-4-yl)amino)-2-cyclopropyl-3,3-difluoro-7-methyl-1,2,3,4-tetrahydro-[1,4]oxazepino[2,3-*c*]quinolin-6(7*H*)-one (20 mg, 0.035 mmol) was purified by chiral column chromatography (Cellulose-4 column (250 × 10 mm, 5 μm) (Phenomenex, Torrence, CA, USA), 100% acetonitrile (0.1% v.v DEA; flow rate 5 mL min^–1^; 100 μL per injection).

The earlier eluting diastereoisomer was arbitrarily assigned as **8a** (5 mg, 0.009 mmol, 25%). HRMS (ESI +ve): found 569.1694 expected 569.1686 for C_25_H_26_ClF_4_N_6_O_3_^+^ [M + H]^+^; 1H NMR (600 MHz, CD_3_OD): δ 8.07 (d, *J* = 2.3 Hz, 1H), 7.98 (s, 1H), 7.94 (dd, *J* = 9.1, 2.3 Hz, 1H), 7.56 (d, *J* = 9.1 Hz, 1H), 4.55–4.39 (m, 2H), 3.92–3.83 (m, 2H + 1H), 3.81–3.75 (m, 2H), 3.73 (s, 3H), 3.37–3.28 (m, 1H), 2.25–2.12 (m, 1H), 1.95–1.83 (m, 1H), 1.49–1.38 (m, 1H), 0.86–0.76 (m, 1H), 0.71–0.58 (m, 2H), 0.42–0.33 (m, 1H).

The later eluting diastereoisomer was arbitrarily assigned as **8b** (4 mg, 0.007 mmol, 20%). HRMS (ESI +ve): found 569.1691 expected 569.1686 for C_25_H_26_ClF_4_N_6_O_3_^+^ [M + H]^+^; 1H NMR (600 MHz, CD_3_OD): δ 8.06 (d, *J* = 2.3 Hz, 1H), 7.99 (s, 1H), 7.93 (dd, *J* = 9.1, 2.3 Hz, 1H), 7.57 (d, *J* = 9.1 Hz, 2H), 4.57–4.39 (m, 2H), 3.96–3.88 (m, 1H), 3.88–3.76 (m, 4H), 3.73 (s, 3H), 3.38–3.28 (m, 1H), 2.25–2.13 (m, 1H), 1.97–1.83 (m, 1H), 1.49–1.38 (m, 1H), 0.86–0.76 (m, 1H), 0.73–0.58 (m, 2H), 0.40–0.32 (m, 1H).

#### (*R*)-(1-Benzyl-4,4-difluoropiperidin-3-yl)methanol (**16a**) and (*S*)-(1-Benzyl-4,4-difluoropiperidin-3-yl)methanol (**16b**)

The commercially available *rac*-(1-benzyl-4,4-difluoro-3-piperidyl)methanol (250 mg) was dissolved to 50 mg/mL in isopropanol and was then purified by SFC (Phenomenex Lux i-Cellulose-5 (21.2 mm × 250 mm, 5 μm), 10:90 isopropanol/CO_2_ (0.2% v/v DEA; flow rate 21 mL min^–1^; 100 μL per injection). The earlier eluting enantiomer was assigned as intermediate **16a** (102 mg), and the later eluting enantiomer was assigned as intermediate **16b** (100 mg). Analyses of the chiral purity of both enantiomers were carried out using SFC (Phenomenex Lux i-Cellulose-5, (4.6 mm × 250 mm, 5 μm), 10:90 isopropanol/CO_2_ (0.2% v/v DEA); flow rate 4 mL min^–1^). **16a**: *ee* = 98.3%; RT 1.70 min **16b**: *ee* = 98.9%; RT 1.91 min.

#### (*R*)-(4,4-Difluoropiperidin-3-yl)methanol (**17a**)

To a solution of (*R*)-1-benzyl-4,4-difluoropiperidin-3-ol (**16a**) (102 mg, 0.42 mmol) in ethanol (8 mL) under argon was added Pd/C (10 wt %; 45 mg, 0.042 mmol). The flask was evacuated and back-filled with hydrogen twice before being stirred at room temperature under a hydrogen balloon for 1 h. The reaction mixture was filtered through Celite (eluent methanol), and the filtrate was concentrated in vacuo to give the title compound as a white solid, which was used without further purification (64 mg, 0.42 mmol, >99%). LCMS (ESI +ve, 2 min) RT 0.21 min; *m*/*z* calcd for C_6_H_12_ F_2_NO^+^ [M + H]^+^: 152.0881, found: 152.0892; δ_H_ (500 MHz, CD_3_OD): 3.92 (dd, *J* = 11.2, 4.0 Hz, 1H), 3.51 (dd, *J* = 11.2, 8.6 Hz, 1H), 3.27–3.21 (m, 1H), 3.07–3.00 (m, 1H), 2.82–2.74 (m, 1H), 2.61 (t, *J* = 12.7, 10.3, 1.7 Hz, 1H), 2.12–1.96 (m, 2H), 1.92–1.78 (m, 1H).

#### (*S*)-(4,4-Difluoropiperidin-3-yl)methanol (**17b**)

To a solution of (*S*)-1-benzyl-4,4-difluoropiperidin-3-ol (**16b**) (100 mg, 0.41 mmol) in ethanol (8 mL) under argon was added Pd/C (10 wt %; 44 mg, 0.041 mmol). The flask was evacuated and back-filled with hydrogen twice before being stirred at room temperature under a hydrogen balloon for 1 h. The reaction mixture was filtered through Celite (eluent methanol), and the filtrate was concentrated in vacuo to give the title compound as a white solid, which was used without further purification (54 mg, 0.35 mmol, 86%). LCMS (ESI +ve, 2 min) RT 0.21 min; *m*/*z*: calcd for C_6_H_12_ F_2_NO^+^ [M + H]^+^ 152.0887; found, 152.0892; δ_H_ (500 MHz, CD_3_OD): 3.92 (dd, *J* = 11.2, 4.0 Hz, 1H), 3.51 (dd, *J* = 11.2, 8.7 Hz, 1H), 3.27–3.20 (m, 1H), 3.07–3.00 (m, 1H), 2.82–2.74 (m, 1H), 2.61 (t, *J* = 12.6, 10.2, 1.7 Hz, 1H), 2.12–1.96 (m, 2H), 1.92–1.78 (m, 1H).

#### (*S*)-10-((5-Chloro-2-((*R*)-4,4-difluoro-3-(hydroxymethyl)piperidin-1-yl)pyrimidin-4-yl)amino)-2-cyclopropyl-3,3-difluoro-7-methyl-1,2,3,4-tetrahydro-[1,4]oxazepino[2,3-*c*]quinolin-6(7*H*)-one (**9a**)

The method is the same as for **8**, using (*R*)-(4,4-difluoropiperidin-3-yl)methanol (**17a**). Purification by preparative HPLC (ACE 5 C18-PFP 250 × 21.2 mm column; 15 min gradient of 40:60 to 25:75 water/methanol (both modified with 0.1% formic acid) at a flow rate of 20 mL/min) was performed to give **9a** (3.0 mg, 0.0051 mmol, 34%). HRMS (ESI +ve): found 583.1835 expected 583.1842 for C_26_H_28_ClF_4_N_6_O_3_^+^ [M + H]^+^; δ_H_ (600 MHz, CD_3_OD): 8.08–8.00 (m, 3H), 7.56 (d, *J* = 9.0 Hz, 1H), 4.55–4.40 (m, 3H), 4.27 (d, *J* = 13.9 Hz, 1H), 3.90 (dd, *J* = 11.1, 4.0 Hz, 1H), 3.74 (s, 3H), 3.53–3.44 (m, 2H), 3.31–3.27 (m, 2H), 2.19–2.07 (m, 1H), 2.06–1.97 (m, 1H), 1.97–1.84 (m, 1H), 1.48–1.38 (m, 1H), 0.85–0.77 (m, 1H), 0.71–0.59 (m, 2H), 0.40–0.33 (m, 1H).

#### (*S*)-10-((5-Chloro-2-((*S*)-4,4-difluoro-3-(hydroxymethyl)piperidin-1-yl)pyrimidin-4-yl)amino)-2-cyclopropyl-3,3-difluoro-7-methyl-1,2,3,4-tetrahydro-[1,4]oxazepino[2,3-*c*]quinolin-6(7*H*)-one (**9b**)

The method is the same as for **8**, using (*S*)-(4,4-difluoropiperidin-3-yl)methanol (**17b**). Purification by preparative HPLC (ACE 5 C18-PFP 250 × 21.2 mm column; 15 min gradient of 40:60 to 25:75 water/methanol (both modified with 0.1% formic acid) at a flow rate of 20 mL/min) was performed to give **9b** (2.2 mg, 0.0038 mmol, 18%). HRMS (ESI +ve): found 583.1852 expected 583.1842 for C_26_H_28_ClF_4_N_6_O_3_^+^ [M + H]^+^; δ_H_ (600 MHz, CD_3_OD): 8.06–8.01 (m, 2H), 8.01 (s, 1H), 7.55 (d, *J* = 9.0 Hz, 1H), 4.58–4.39 (m, 3H), 4.34–4.27 (m, 1H), 3.91 (dd, *J* = 11.1, 4.0 Hz, 1H), 3.73 (s, 3H), 3.49 (dd, *J* = 11.1, 9.4 Hz, 1H), 3.46–3.38 (m, 1H), 3.37–3.24 (m, 2H), 2.20–2.08 (m, 1H), 2.07–1.97 (m, 1H), 1.97–1.83 (m, 1H), 1.46–1.37 (m, 1H), 0.85–0.77 (m, 1H), 0.72–0.66 (m, 1H), 0.66–0.59 (m, 1H), 0.40–0.33 (m, 1H).

#### *N*-((3*R*,5*S*)-1-(5-Chloro-4-(((*S*)-2-cyclopropyl-3,3-difluoro-7-methyl-6-oxo-1,2,3,4,6,7-hexahydro-[1,4]oxazepino[2,3-*c*]quinolin-10-yl)amino)pyrimidin-2-yl)-5-methylpiperidin-3-yl)acetamide (**10a**)

##### Step 1: *tert*-Butyl (3*R*,5*S*)-3-Acetamido-5-methylpiperidine-1-carboxylate (**18a**)

A mixture of *tert*-butyl (3*R*,5*S*)-3-amino-5-methyl-piperidine-1-carboxylate (135.00 mg, 0.63 mmol), DIPEA (143 μL, 0.82 mmol), and acetic anhydride (66 μL, 0.69 mmol) was dissolved in DCM (5 mL) and stirred at rt for 2 h. The reaction mixture was diluted with DCM (10 mL) and washed with water (2 × 5 mL) and saturated brine solution (5 mL). The organic layer was dried over MgSO_4_, and the solvent was removed at reduced pressure to yield **18a** as a yellow oil (120 mg, 0.47 mmol, 74%). δ_H_ (600 MHz, CDCl_3_): 5.94 (s, 1H), 4.31–4.15 (m, 1H), 4.14–3.87 (m, 1H), 3.81 (m, 1H), 2.31 (t, *J* = 11.8 Hz, 1H), 2.19–2.11 (m, 1H), 2.04–2.00 (m, 1H), 1.97 (s, 3H), 1.66 (m, 1H), 1.44 (s, 9H), 0.93–0.86 (m, 4H).

##### Step 2: *N*-((3*R*,5*S*)-5-Methylpiperidin-3-yl)acetamide (**19a**)

A mixture of *tert*-butyl (3*R*,5*S*)-3-acetamido-5-methylpiperidine-1-carboxylate (**18a**) (120 mg, 0.47 mmol) and trifluoroacetic acid (122 μL, 1.59 mmol) in DCM (1 mL) was stirred at rt for 18 h. The solvent was removed at reduced pressure and purification using an SCX-2 column gave **19a** as a colourless oil (73 mg, 0.47 mmol, >99%). δ_H_ (500 MHz, CD_3_OD): 3.79–3.70 (m, 1H), 3.13–2.98 (m, 1H), 2.95–2.84 (m, 1H), 2.18 (dd, *J* = 12.1, 11.1 Hz, 1H), 2.05 (dd, *J* = 12.6, 11.3 Hz, 1H), 1.97–1.92 (m, 1H), 1.91 (s, 3H), 1.70–1.56 (m, 1H), 0.97 (q, *J* = 12.1 Hz, 1H), 0.89 (d, *J* = 6.6 Hz, 3H).

##### Step 3: *N*-((3*R*,5*S*)-1-(5-Chloro-4-(((*S*)-2-cyclopropyl-3,3-difluoro-7-methyl-6-oxo-1,2,3,4,6,7-hexahydro-[1,4]oxazepino[2,3-*c*]quinolin-10-yl)amino)pyrimidin-2-yl)-5-methylpiperidin-3-yl)acetamide (**10a**)

The method is the same as for **8**, using *N*-((3*R*,5*S*)-5-methylpiperidin-3-yl)acetamide (**19a**). Purification by reverse-phase chromatography eluting from 40 to 90% methanol in water (both modified with 0.1% formic acid), followed by further purification using an SCX-2 column, was performed to give **10a** (4.5 mg, 0.0077 mmol, 23%). HRMS (ESI +ve): found 588.2302 expected 588.2302 for C_28_H_33_ClF_2_N_7_O_3_ [M + H]^+^; δ_H_ (600 MHz, CD_3_OD): 8.08 (d, *J* = 9.1 Hz, 1H), 7.96 (s, 1H), 7.94 (s, 1H), 7.54 (d, *J* = 9.1 Hz, 1H), 4.76–4.71 (m, 1H), 4.56–4.37 (m, 3H), 3.79–3.74 (m, 1H), 3.73 (s, 3H), 3.38–3.27 (m, 1H), 2.44 (dd, *J* = 12.6, 11.1 Hz, 1H), 2.32 (dd, *J* = 13.1, 11.4 Hz, 1H), 2.03–1.98 (m, 1H), 1.96 (s, 3H), 1.71–1.61 (m, 1H), 1.45–1.38 (m, 1H), 1.10 (q, *J* = 12.1 Hz, 1H), 0.94 (d, *J* = 6.6 Hz, 3H), 0.84–0.78 (m, 1H), 0.72–0.65 (m, 1H), 0.65–0.58 (m, 1H), 0.41–0.33 (m, 1H).

#### *N*-((3*S*,5*R*)-1-(5-Chloro-4-(((*S*)-2-cyclopropyl-3,3-difluoro-7-methyl-6-oxo-1,2,3,4,6,7-hexahydro-[1,4]oxazepino[2,3-*c*]quinolin-10-yl)amino)pyrimidin-2-yl)-5-methylpiperidin-3-yl)acetamide (**10b**)

##### Step 1: *tert*-Butyl (3*S*,5*R*)-3-Acetamido-5-methylpiperidine-1-carboxylate (**18b**)

A mixture of *tert*-butyl (3*R*,5*S*)-3-amino-5-methyl-piperidine-1-carboxylate (125.00 mg, 0.58 mmol), DIPEA (132 μL, 0.82 mmol), and acetic anhydride (61 μL, 0.66 mmol) in DCM (5 mL) was stirred at rt for 2 h. The reaction mixture was diluted with DCM (10 mL) and washed with water (2 × 5 mL) and saturated brine solution (5 mL). The organic layer was dried over MgSO_4_, and the solvent was removed at reduced pressure to yield **18b** as a yellow oil (83 mg, 0.32 mmol, 74%). δ_H_ (600 MHz, CDCl_3_): δ 6.05 (s, 1H), 4.23 (d, *J* = 12.4 Hz, 1H), 4.11–3.98 (m, 1H), 3.86–3.73 (m, 1H), 2.31 (t, *J* = 11.8 Hz, 1H), 2.20–2.10 (m, 2H), 2.03–1.99 (m, 1H), 1.96 (s, 3H), 1.71–1.60 (m, 1H), 1.46 (s, 9H), 0.95–0.84 (m, 4H).

##### Step 2: *N*-((3*S*,5*R*)-5-Methylpiperidin-3-yl)acetamide (**19b**)

A mixture of *tert*-butyl (3*S*,5*R*)-3-acetamido-5-methylpiperidine-1-carboxylate (**18b**) (83 mg, 0.32 mmol) and trifluoroacetic acid (84 μL, 1.10 mmol) in DCM (1 mL) was stirred at rt for 18 h. The solvent was removed at reduced pressure, and purification using an SCX-2 column gave **19b** as a colourless oil (44 mg, 0.28 mmol, 87%). δ_H_ (500 MHz, CD_3_OD): 3.80–3.66 (m, 1H), 3.14–2.99 (m, 1H), 2.95–2.81 (m, 1H), 2.17 (dd, *J* = 12.1, 11.0 Hz, 1H), 2.03 (dd, *J* = 12.6, 11.3 Hz, 1H), 1.98–1.91 (m, 1H), 1.90 (s, 3H), 1.72–1.56 (m, 1H), 0.96 (q, *J* = 12.1 Hz, 1H), 0.88 (d, *J* = 6.6 Hz, 3H).

##### Step 3: *N*-((3*S*,5*R*)-1-(5-Chloro-4-(((*S*)-2-cyclopropyl-3,3-difluoro-7-methyl-6-oxo-1,2,3,4,6,7-hexahydro-[1,4]oxazepino[2,3-*c*]quinolin-10-yl)amino)pyrimidin-2-yl)-5-methylpiperidin-3-yl)acetamide (**10b**)

The method is the same as for **8**, using *N*-((3*S*,5*R*)-5-methylpiperidin-3-yl)acetamide (**19b**). Purification by reverse-phase chromatography eluting from 40 to 90% methanol in water (both modified with 0.1% formic acid), followed by further purification using an SCX-2 column, was performed to give **10b** (5.0 mg, 0.0085 mmol, 23%). HRMS (ESI +ve): found 588.2302 expected 588.2302 for C_28_H_33_ClF_2_N_7_O_3_ [M + H]^+^; δ_H_ (600 MHz, CD_3_OD): 8.08 (d, *J* = 9.1 Hz, 1H), 7.97–7.92 (m, 2H), 7.54 (d, *J* = 9.1 Hz, 1H), 4.78–4.67 (m, 1H), 4.57–4.34 (m, 3H), 3.80–3.73 (m, 1H), 3.73 (s, 3H), 3.40–3.26 (m, 1H), 2.43 (dd, *J* = 12.6, 11.2 Hz, 1H), 2.31 (dd, *J* = 13.1, 11.4 Hz, 1H), 1.97 (m, 4H), 1.66 (m, 1H), 1.46–1.36 (m, 1H), 1.14–1.05 (m, 1H), 0.94 (d, *J* = 6.5 Hz, 3H), 0.83–0.77 (m, 1H), 0.72–0.65 (m, 1H), 0.65–0.59 (m, 1H), 0.41–0.35 (m, 1H).

#### (*S*)-10-((5-Chloro-2-((3*R*,5*S*)-3-(dimethylamino)-5-methylpiperidin-1-yl)pyrimidin-4-yl)amino)-2-cyclopropyl-3,3-difluoro-7-methyl-1,2,3,4-tetrahydro-[1,4]oxazepino[2,3-*c*]quinolin-6(7*H*)-one (**11a**)

##### Step 1: *tert*-Butyl (3*R*,5*S*)-3-(Dimethylamino)-5-methylpiperidine-1-carboxylate (**20a**)

A mixture of *tert*-butyl (3*R*,5*S*)-3-amino-5-methyl-piperidine-1-carboxylate (65 mg, 0.30 mmol), formaldehyde (37% v/v in water) (452 μL, 6.07 mmol), and sodium cyanoborohydride (80 mg, 1.27 mmol) was stirred at 50 °C for 18 h. The solvent was removed at reduced pressure, and the residue was dissolved in EtOAc (15 mL) and washed with saturated NaHCO_3_ solution (2 × 5 mL) and saturated brine solution (5 mL). The organic layer was dried over MgSO_4_, and the solvent was removed at reduced pressure to yield **20a** as a yellow oil (74 mg, 0.30 mmol, >99%). δ_H_ (500 MHz, CDCl_3_): 4.36–4.11 (m, 1H), 4.00–3.82 (m, 1H), 2.41–2.32 (m, 1H), 2.27 (s, 6H), 2.21–2.14 (m, 1H), 2.13–2.07 (m, 1H), 1.96–1.91 (m, 1H), 1.60–1.47 (m, 1H), 1.38 (s, 9H), 0.91 (q, *J* = 12.1 Hz, 1H), 0.85 (d, *J* = 6.6 Hz, 3H).

##### Step 2: (3*R*,5*S*)-*N*,*N*,5-Trimethylpiperidin-3-amine (**21a**)

A mixture of *tert*-butyl (3*R*,5*S*)-3-(dimethylamino)-5-methylpiperidine-1-carboxylate (**20a**) (74 mg, 0.30 mmol) and trifluoroacetic acid (100 μL, 3.41 mmol) in DCM (1 mL) was stirred at rt for 18 h. The solvent was removed at reduced pressure, and purification using an SCX-2 column gave **21a** as a colourless oil (25 mg, 0.18 mmol, 58%). δ_H_ (500 MHz, CD_3_OD): 3.21–3.14 (m, 1H), 2.96–2.86 (m, 1H), 2.36–2.30 (m, 2H), 2.32 (s, 6H), 2.12–1.99 (m, 2H), 1.67–1.53 (m, 1H), 1.02–0.94 (m, 1H), 0.92 (d, *J* = 6.6 Hz, 3H).

##### Step 3: (*S*)-10-((5-Chloro-2-((3*R*,5*S*)-3-(dimethylamino)-5-methylpiperidin-1-yl)pyrimidin-4-yl)amino)-2-cyclopropyl-3,3-difluoro-7-methyl-1,2,3,4-tetrahydro-[1,4]oxazepino[2,3-c]quinolin-6(7*H*)-one (**11a**)

The method is the same as for **8**, using (3*R*,5*S*)-*N*,*N*,5-trimethylpiperidin-3-amine (**21a**). Purification by reverse-phase chromatography eluting from 40 to 90% methanol in water (both modified with 0.1% formic acid), followed by further purification using an SCX-2 column, was performed to give **11a** (9.4 mg, 0.016 mmol, 26%). HRMS (ESI +ve): found 574.2510 expected 574.2509 for C_28_H_35_ClF_2_N_7_O_2_ [M + H]^+^; δ_H_ (600 MHz, DMSO-*d*_6_): 8.84 (s, 1H), 8.10 (d, *J* = 2.3 Hz, 1H), 8.02 (s, 1H), 7.70 (dd, *J* = 8.9, 2.2 Hz, 1H), 7.40 (d, *J* = 9.0 Hz, 1H), 6.27 (s, 1H), 4.63 (s, 1H), 4.55–4.24 (m, 3H), 3.55 (s, 3H), 3.24–3.16 (m, 1H), 2.47–2.34 (m, 2H), 2.22 (t, *J* = 12.2 Hz, 1H), 2.18–2.04 (m, 6H), 1.89 (d, *J* = 12.3 Hz, 1H), 1.50–1.41 (m, 1H), 1.37–1.28 (m, 1H), 1.05–0.95 (m, 1H), 0.84 (d, *J* = 6.5 Hz, 3H), 0.75–0.67 (m, 1H), 0.56–0.47 (m, 2H), 0.40–0.28 (m, 1H).

#### (*S*)-10-((5-Chloro-2-((3*S*,5*R*)-3-(dimethylamino)-5-methylpiperidin-1-yl)pyrimidin-4-yl)amino)-2-cyclopropyl-3,3-difluoro-7-methyl-1,2,3,4-tetrahydro-[1,4]oxazepino[2,3-*c*]quinolin-6(7*H*)-one (**11b**)

##### Step 1: *tert*-Butyl (3*S*,5*R*)-3-(Dimethylamino)-5-methylpiperidine-1-carboxylate (**20b**)

A mixture of *tert*-butyl (3*S*,5*R*)-3-amino-5-methyl-piperidine-1-carboxylate (82 mg, 0.38 mmol), formaldehyde (37% v/v in water) (570 μL, 7.65 mmol), and sodium cyanoborohydride (101 mg, 1.61 mmol) was stirred at 50 °C for 18 h. The solvent was removed at reduced pressure, and the residue was dissolved in EtOAc (15 mL) and washed with saturated NaHCO_3_ solution (2 × 5 mL) and saturated brine solution (5 mL). The organic layer was dried over MgSO_4_, and the solvent was removed at reduced pressure to yield **20b** as a yellow oil (93 mg, 0.38 mmol, >99%). δ_H_ (500 MHz, CDCl_3_): 4.44–4.18 (m, 1H), 4.06–3.86 (m, 1H), 2.55–2.36 (m, 1H), 2.32 (s, 6H), 2.27–2.19 (m, 1H), 2.18–2.10 (m, 1H), 2.01–1.93 (m, 1H), 1.61–1.54 (m, 1H), 1.43 (s, 9H), 0.95 (q, *J* = 12.0 Hz, 1H), 0.90 (d, *J* = 6.6 Hz, 3H).

##### Step 2: (3*S*,5*R*)-*N*,*N*,5-Trimethylpiperidin-3-amine (**21b**)

A mixture of *tert*-butyl (3*S*,5*R*)-3-(dimethylamino)-5-methylpiperidine-1-carboxylate (**20b**) (93 mg, 0.38 mmol) and trifluoroacetic acid (100 μL, 3.41 mmol) in DCM (1 mL) was stirred at rt for 18 h. The solvent was removed at reduced pressure, and purification using an SCX-2 column gave **21b** as a colourless oil (55 mg, 0.38 mmol, >99%). δ_H_ (500 MHz, CD_3_OD): 3.22–3.12 (m, 1H), 2.96–2.83 (m, 1H), 2.35–2.31 (m, 2H), 2.31 (s, 6H), 2.09–1.98 (m, 2H), 1.67–1.51 (m, 1H), 1.03–0.94 (m, 1H), 0.92 (d, *J* = 6.6 Hz, 3H).

##### Step 3: (*S*)-10-((5-Chloro-2-((3*S*,5*R*)-3-(dimethylamino)-5-methylpiperidin-1-yl)pyrimidin-4-yl)amino)-2-cyclopropyl-3,3-difluoro-7-methyl-1,2,3,4-tetrahydro-[1,4]oxazepino[2,3-*c*]quinolin-6(7*H*)-one (**11b**)

The method is the same as for **8**, using (3*S*,5*R*)-*N*,*N*,5-trimethylpiperidin-3-amine (**21b**). Purification by reverse-phase chromatography eluting from 40 to 90% methanol in water (both modified with 0.1% formic acid), followed by further purification using an SCX-2 column, was performed to give **11b** (4.3 mg, 0.0075 mmol, 26%). HRMS (ESI +ve): found 574.2503 expected 574.2509 for C_28_H_35_ClF_2_N_7_O_2_ [M + H]^+^; δ_H_ (600 MHz, DMSO-*d*_6_): 8.85 (s, 1H), 8.10 (s, 1H), 8.03 (s, 1H), 7.72 (dd, *J* = 9.0, 2.2 Hz, 1H), 7.41 (d, *J* = 9.0 Hz, 1H), 6.25 (s, 1H), 4.53–4.29 (m, 4H), 3.56 (s, 3H), 3.30–3.14 (m, 1H), 2.65–2.44 (m, 1H), 2.35–2.11 (m, 7H), 1.92 (d, *J* = 11.7 Hz, 1H), 1.48 (m, 1H), 1.38–1.26 (m, 1H), 1.12–0.99 (m, 1H), 0.85 (d, *J* = 6.4 Hz, 3H), 0.75–0.68 (m, 1H), 0.55–0.45 (m, 2H), 0.39–0.29 (m, 1H).

#### (3*R*,5*S*)-1-Benzyl-5-methylpiperidin-3-ol (**22a**) and (3*S*,5*R*)-1-Benzyl-5-methylpiperidin-3-ol (**22b**)

The commercially available *rac*-1-benzyl-5-methylpiperidin-3-ol hydrogen chloride (13 g) was dissolved to 90 mg/mL in 10:1 MeOH/DCM and was then purified by SFC (Lux A1 (30 mm × 250 mm, 5 μm), 10:90 MeOH/CO_2_ (0.2% v/v DEA; flow rate 100 mL min^–1^). The earlier eluting enantiomer was identified as intermediate **22a** (4.9 g), and the later eluting enantiomer was identified as intermediate **22b** (4.2 g). Analyses of the chiral purity of both enantiomers were carried out using SFC (YMC Chiral ART Amylose-C (4.6 mm × 250 mm, 5 μm), 10:90 MeOH/CO_2_ (0.2% v/v DEA); flow rate 4 mL min^–1^). **22a**: *ee* = 99.6%; RT 2.03 min (Figure S6). **22b**: *ee* = 98.7%; RT 2.80 min. The absolute stereochemistry ((3*S*,5*R*)-1-benzyl-5-methylpiperidin-3-ol) of **22b** was assigned from its small-molecule X-ray crystal structure (Supporting Information Supplementary experimental 2.1, Table S4 and Figure S7).^20^

#### (3*R*,5*S*)-5-Methylpiperidin-3-ol (**23a**)

To a solution of (3*R*,5*S*)-1-benzyl-5-methylpiperidin-3-ol (**22a**, 1.9 g, 9.25 mmol) in ethanol (100 mL) under argon was added Pd/C (10 wt %; 452 mg, 0.43 mmol). The flask was evacuated and back-filled with hydrogen twice before being stirred at room temperature under a hydrogen balloon for 3 h. The reaction mixture was filtered through Celite (eluent methanol), and the filtrate was concentrated in vacuo to give the title compound (64 mg, 0.56 mmol, >99%) as a white solid, which was used without further purification. δ_H_ (500 MHz, CD_3_OD): δ 3.60–3.53 (m, 1H), 3.09 (dddd, *J* = 11.8, 4.7, 2.2, 1.1 Hz, 1H), 2.93–2.83 (m, 1H), 2.22 (dd, *J* = 11.8, 10.4 Hz, 1H), 2.13–1.98 (m, 2H), 1.70–1.56 (m, 1H), 0.96 (td, *J* = 12.1, 11.0 Hz, 1H), 0.91 (d, *J* = 6.6 Hz, 3H).

#### (3*S*,5*R*)-5-Methylpiperidin-3-ol (**23b**)

To a solution of (3*S*,5*R*)-1-benzyl-5-methylpiperidin-3-ol (**22b**, 62 mg, 0.30 mmol) in ethanol (5 mL) under argon was added Pd/C (10 wt %; 32 mg, 0.030 mmol). The flask was evacuated and back-filled with hydrogen twice before being stirred at room temperature under a hydrogen balloon for 3 h. The reaction mixture was filtered through Celite (eluent methanol), and the filtrate was concentrated in vacuo to give the title compound (33 mg, 0.29 mmol, 95%) as a white solid, which was used without further purification. δ_H_ (500 MHz, DMSO-*d*_6_): 4.49 (d, *J* = 4.7 Hz, 1H), 3.32–3.25 (m, 1H), 2.90 (dddd, *J* = 11.4, 4.6, 2.1, 1.0 Hz, 1H), 2.76–2.67 (m, 1H), 1.99 (dd, *J* = 11.5, 10.0 Hz, 1H), 1.88–1.78 (m, 2H), 1.48–1.35 (m, 1H), 0.84–0.73 (m, 4H).

#### (*S*)-10-((5-Chloro-2-((3*R*,5*S*)-3-hydroxy-5-methylpiperidin-1-yl)pyrimidin-4-yl)amino)-2-cyclopropyl-3,3-difluoro-7-methyl-1,2,3,4-tetrahydro-[1,4]oxazepino[2,3-*c*]quinolin-6(7*H*)-one (**12a**)

A mixture of (*S*)-2-cyclopropyl-10-((2,5-dichloropyrimidin-4-yl)amino)-3,3-difluoro-7-methyl-1,2,3,4-tetrahydro-[1,4]oxazepino[2,3-*c*]quinolin-6(7*H*)-one (**7**) (1.7 g, 3.63 mmol), (3*R*,5*S*)-5-methylpiperidin-3-ol (**23a**) (500 mg, 4.34 mmol), and DIPEA (1 mL, 5.74 mmol) in acetonitrile (30 mL) was at 80 °C for 18 h. The resulting mixture was purified by flash column chromatography (0–10% MeOH in DCM) to give **CCT373566** (**12a**) (1.5 g, 2.75 mmol, 75%). HRMS (ESI +ve): found 547.2004 expected 547.2030 for C_26_H_30_ClF_2_N_6_O_3_ [M + H]^+^; δ_H_ (600 MHz, CD_3_OD): 8.02 (d, *J* = 2.3 Hz, 1H), 8.00 (dd, *J* = 9.0, 2.3 Hz, 1H), 7.95 (s, 1H), 7.56 (d, *J* = 9.1 Hz, 1H), 4.76–4.68 (m, 1H), 4.55–4.35 (m, 3H), 3.73 (s, 3H), 3.60–3.47 (m, 1H), 3.38–3.27 (m, 1H), 2.47 (dd, *J* = 12.4, 10.5 Hz, 1H), 2.29 (dd, *J* = 13.0, 11.4 Hz, 1H), 2.13–2.02 (m, 1H), 1.67–1.55 (m, 1H), 1.49–1.34 (m, 1H), 1.07 (q, *J* = 11.9 Hz, 1H), 0.94 (d, *J* = 6.6 Hz, 3H), 0.86–0.78 (m, 1H), 0.75–0.66 (m, 1H), 0.66–0.59 (m, 1H), 0.45–0.34 (m, 1H).

#### (*S*)-10-((5-Chloro-2-((3*S*,5*R*)-3-hydroxy-5-methylpiperidin-1-yl)pyrimidin-4-yl)amino)-2-cyclopropyl-3,3-difluoro-7-methyl-1,2,3,4-tetrahydro-[1,4]oxazepino[2,3-*c*]quinolin-6(7*H*)-one (**12b**)

The method is the same as for **8**, using (3*S*,5*R*)-5-methylpiperidin-3-ol (**23b**). Purification by reverse-phase chromatography eluting from 30 to 100% methanol in water (both modified with 0.1% formic acid) was performed to give CCT373567 (**12b**) (7.9 mg, 0.014 mmol, 34%). HRMS (ESI +ve): found 547.2014 expected 547.2030 for C_26_H_30_ClF_2_N_6_O_3_ [M + H]^+^; δ_H_ (600 MHz, DMSO-*d*_6_): 8.78 (s, 1H), 8.11 (s, 1H), 8.02 (s, 1H), 7.75 (d, *J* = 9.0 Hz, 1H), 7.44 (d, *J* = 9.0 Hz, 1H), 6.20 (d, *J* = 4.2 Hz, 1H), 4.88–4.80 (m, 1H), 4.57 (s, 1H), 4.50–4.25 (m, 3H), 3.57 (s, 3H), 3.36–3.18 (m, 2H), 2.32 (dd, *J* = 12.3, 10.4 Hz, 1H), 2.18 (t, *J* = 12.1 Hz, 1H), 1.91 (q, *J* = 5.7, 5.0 Hz, 1H), 1.55–1.44 (m, 1H), 1.36–1.28 (m, 1H), 0.94 (q, *J* = 11.8 Hz, 1H), 0.81 (d, *J* = 6.7 Hz, 3H), 0.74–0.67 (m, 1H), 0.56–0.47 (m, 2H), 0.38–0.30 (m, 1H).

#### (*S*)-10-((5-Chloro-2-((3*R*,5*R*)-3-hydroxy-5-methylpiperidin-1-yl)pyrimidin-4-yl)amino)-2-cyclopropyl-3,3-difluoro-7-methyl-1,2,3,4-tetrahydro-[1,4]oxazepino[2,3-*c*]quinolin-6(7*H*)-one (**13a**)

##### Step 1: (3*R*,7a*S*)-3-Phenyltetrahydro-3*H*,5*H*-pyrrolo[1,2-*c*]oxazol-5-one (**24a**)^17^

To a solution of (*S*)-5-(hydroxymethyl)pyrrolidin-2-one (2.00 g, 17.37 mmol) in toluene (60 mL) were added benzaldehyde (2.30 mL, 22.58 mmol) and *p*-toluenesulfonic acid monohydrate (33 mg, 0.17 mmol). The reaction mixture was heated at 120 °C for 16 h, and the water formed during the reaction mixture was separated out using a Dean–Stark condenser. The reaction mixture was allowed to cool to rt and washed with 5% NaHCO_3_ solution (100 mL), 20% NaHSO_3_ solution (2 × 50 mL) and brine (50 mL). The solvent was removed at reduced pressure, and the residue was purified by flash column chromatography (Biotage 50 g KP-sil, 5–50% EtOAc in cyclohexane) to give **24a** as a yellow oil (2.19 g, 10.8 mmol, 61%). δ_H_ (500 MHz, CDCl_3_): 7.51–7.43 (m, 2H), 7.43–7.31 (m, 3H), 6.40–6.33 (m, 1H), 4.33–4.23 (m, 1H), 4.22–4.13 (m, 1H), 3.56–3.46 (m, 1H), 2.91–2.77 (m, 1H), 2.64–2.53 (m, 1H), 2.47–2.34 (m, 1H), 2.04–1.91 (m, 1H).

##### Step 2: (3*R*,6*R*,7a*S*)-6-Methyl-3-phenyltetrahydro-3*H*,5*H*-pyrrolo[1,2-*c*]oxazol-5-one (**25a**)

To a solution of (3*R*,7a*S*)-3-phenyltetrahydro-3*H*,5*H*-pyrrolo[1,2-*c*]oxazol-5-one (**24a**) (4.0 g, 19.48 mmol) in THF (100 mL) at −78 °C was added LDA (2 M in THF) (9.74 mL, 19.48 mmol), and the reaction mixture was allowed to warm to rt over 30 min. The resulting mixture was cooled to −78 °C, and iodomethane (1.21 mL, 19.48 mmol) was added dropwise. The reaction mixture was allowed to warm to rt, stirred for 4 h, and then quenched upon the addition of water (20 mL). The mixture was extracted with EtOAc (3 × 75 mL). The organic layers were combined and washed with sat. brine (50 mL), and the solvent was removed at reduced pressure. The resulting yellow oil was purified by flash column chromatography (Biotage 340 g KP-sil, 12–100% EtOAc in cyclohexane) to yield the minor isomer **25a** as a yellow oil (597 mg, 2.75 mmol, 14%). δ_H_ (500 MHz, CDCl_3_): 7.50–7.44 (m, 2H), 7.43–7.30 (m, 3H), 6.34 (s, 1H), 4.25 (dd, *J* = 8.0, 6.3 Hz, 1H), 4.18–4.06 (m, 1H), 3.47–3.40 (m, 1H), 2.80–2.69 (m, 1H), 2.21 (ddd, *J* = 13.5, 9.4, 4.1 Hz, 1H), 2.08–1.96 (m, 1H), 1.37 (d, *J* = 7.4 Hz, 3H).

##### Step 3: ((2*S*,4*R*)-1-Benzyl-4-methylpyrrolidin-2-yl)methanol (**26a**)

To a solution of (3*R*,6*R*,7a*S*)-6-methyl-3-phenyltetrahydro-3*H*,5*H*-pyrrolo[1,2-*c*]oxazol-5-one (**25a**) (597 mg, 2.75 mmol) in THF (25 mL) was added LiAlH_4_ (1 M in THF) (6.9 mL, 6.87 mmol), and the mixture was stirred at rt for 4 h. The reaction mixture was cooled to 0 °C and quenched with the dropwise addition of water (0.6 mL), aqueous NaOH (2 M) (1 mL), and water (2.7 mL). The resulting suspension was diluted with Et_2_O (10 mL) and stirred at rt for 30 min. Na_2_SO_4_ and Celite were added, and the slurry was filtered and washed with Et_2_O (100 mL). The filtrate was concentrated at reduced pressure to give **26a** as a colourless oil (522 mg, 2.55 mmol, 92%). δ_H_ (500 MHz, CDCl_3_): 7.38–7.20 (m, 5H), 3.96 (d, *J* = 12.9 Hz, 1H), 3.66–3.59 (m, 1H), 3.44–3.34 (m, 2H), 3.06–3.00 (m, 1H), 2.88–2.80 (m, 1H), 2.21–2.10 (m, 1H), 2.04–1.91 (m, 2H), 1.60–1.50 (m, 1H), 0.97 (d, *J* = 6.5 Hz, 3H).

##### Step 4: (3*R*,5*R*)-1-Benzyl-5-methylpiperidin-3-ol (**27a**)

A solution of ((2*S*,4*R*)-1-benzyl-4-methylpyrrolidin-2-yl)methanol (**26a**) (522 mg, 2.54 mmol) in THF (15 mL) was cooled to −78 °C, and to this was added trifluoroacetic acid anhydride (424 μL, 3.05 mmol). The mixture was stirred at −78 °C for 45 min and then allowed to warm to rt and stirred for a further 45 min. The reaction mixture was cooled to −78 °C, triethylamine (1.42 mL, 10.12 mmol) was added, and the resulting solution was stirred at −78 °C for 15 min before heating at reflux for 16 h. The reaction mixture was cooled to rt, and aqueous NaOH solution (2 M, 0.75 mL, 1.49 mmol) was added, and the solution was stirred for 3 h. DCM (20 mL) was added, and the solution was washed with sat. NH_4_Cl solution (15 mL), sat. NaHCO_3_ solution (15 mL), and sat. brine (15 mL). The organic layer was dried over MgSO_4_, and the solvent was removed under reduced pressure. The resulting residue was purified by flash column chromatography (Biotage 50 g KP-sil, 20–80% EtOAc in cyclohexane) to give **27a** as a yellow oil (246 mg, 1.20 mmol, 42%). δ_H_ (500 MHz, CDCl_3_): 7.38–7.20 (m, 5H), 3.89 (qd, *J* = 3.4, 1.7 Hz, 1H), 3.52 (s, 2H), 2.93–2.83 (m, 1H), 2.80 (ddt, *J* = 11.0, 3.9, 1.8 Hz, 1H), 2.12 (dd, *J* = 11.3, 1.6 Hz, 1H), 2.05–1.95 (m, 1H), 1.86 (dtt, *J* = 13.4, 3.7, 1.8 Hz, 1H), 1.63 (t, *J* = 10.9 Hz, 1H), 1.06 (ddd, *J* = 13.4, 12.1, 2.7 Hz, 1H), 0.86 (d, *J* = 6.7 Hz, 3H).

##### Step 5: (3*R*,5*R*)-5-Methylpiperidin-3-ol (**28a**)

To a solution of (3*R*,5*R*)-1-benzyl-5-methylpiperidin-3-ol (**27a**) (247 mg, 1.20 mmol) in ethanol (5 mL) under argon was added Pd/C (10 wt %; 128 mg, 0.12 mmol). The flask was evacuated and back-filled with hydrogen twice before being stirred at room temperature under a hydrogen balloon for 3 h. The reaction mixture was filtered through Celite (eluent methanol), and the filtrate was concentrated in vacuo to give the title compound (153 mg, 1.20 mmol, >99%) as a white solid, which was used without further purification. δ_H_ (500 MHz, CD_3_OD): 3.82–3.74 (m, 1H), 2.92–2.82 (m, 1H), 2.82–2.71 (m, 1H), 2.66–2.57 (m, 1H), 2.18–2.08 (m, 1H), 1.96–1.84 (m, 1H), 1.81–1.72 (m, 1H), 1.33–1.21 (m, 1H), 0.81 (d, *J* = 6.8 Hz, 3H).

##### Step 6: (*S*)-10-((5-Chloro-2-((3*R*,5*R*)-3-hydroxy-5-methylpiperidin-1-yl)pyrimidin-4-yl)amino)-2-cyclopropyl-3,3-difluoro-7-methyl-1,2,3,4-tetrahydro-[1,4]oxazepino[2,3-c]quinolin-6(7*H*)-one (**13a**)

The method is the same as for **8**, using (3*R*,5*R*)-5-methylpiperidin-3-ol (**28a**). Purification by reverse-phase chromatography eluting from 30 to 100% methanol in water (both modified with 0.1% formic acid) was performed to give **13a** (11.7 mg, 0.021 mmol, 50%). HRMS (ESI +ve): found 547.2024 expected 547.2030 for C_26_H_30_ClF_2_N_6_O_3_ [M + H]^+^; δ_H_ (600 MHz, CD_3_OD): 8.15 (d, *J* = 2.2 Hz, 1H), 7.95 (dd, *J* = 9.1, 2.3 Hz, 1H), 7.93 (s, 1H), 7.56 (d, *J* = 9.1 Hz, 1H), 4.58–4.40 (m, 2H), 4.37–4.22 (m, 2H), 4.00–3.91 (m, 1H), 3.74 (s, 3H), 3.35–3.24 (m, 1H), 3.19 (dd, *J* = 13.6, 2.4 Hz, 1H), 2.70 (dd, *J* = 13.0, 10.0 Hz, 1H), 2.14–2.01 (m, 1H), 1.91–1.77 (m, 1H), 1.47–1.39 (m, 2H), 0.90 (d, *J* = 6.7 Hz, 3H), 0.84–0.76 (m, 1H), 0.70–0.56 (m, 2H), 0.41–0.31 (m, 1H).

#### (*S*)-10-((5-Chloro-2-((3*S*,5*S*)-3-hydroxy-5-methylpiperidin-1-yl)pyrimidin-4-yl)amino)-2-cyclopropyl-3,3-difluoro-7-methyl-1,2,3,4-tetrahydro-[1,4]oxazepino[2,3-*c*]quinolin-6(7*H*)-one (**13b**)

##### Step 1: (3*S*,7a*R*)-3-Phenyltetrahydro-3*H*,5*H*-pyrrolo[1,2-*c*]oxazol-5-one (**24b**)^17^

To a solution of (*R*)-5-(hydroxymethyl)pyrrolidin-2-one (2.09 g, 18.15 mmol) in toluene (60 mL) were added benzaldehyde (2.50 mL, 23.60 mmol) and *p*-toluenesulfonic acid monohydrate (35 mg, 0.18 mmol). The reaction mixture was heated at 120 °C for 16 h, and the water formed during the reaction mixture was separated out using a Dean–Stark condenser. The reaction mixture was allowed to cool to rt and washed with 5% NaHCO_3_ solution (100 mL), 20% NaHSO_3_ solution (2 × 50 mL), and brine (50 mL). The solvent was removed at reduced pressure, and the residue was purified by flash column chromatography (Biotage 50 g KP-sil, 5–50% EtOAc in cyclohexane) to give **24b** as a yellow oil (1.74 g, 8.57 mmol, 47%). δ_H_ (500 MHz, CDCl_3_): 7.49–7.45 (m, 2H), 7.41–7.32 (m, 3H), 6.35 (s, 1H), 4.31–4.22 (m, 1H), 4.22–4.10 (m, 1H), 3.51 (t, *J* = 8.1 Hz, 1H), 2.93–2.78 (m, 1H), 2.63–2.52 (m, 1H), 2.48–2.34 (m, 1H), 2.04–1.91 (m, 1H).

##### Step 2: (3*S*,6*S*,7a*R*)-6-Methyl-3-phenyltetrahydro-3*H*,5*H*-pyrrolo[1,2-*c*]oxazol-5-one (**25b**)

To a solution of (3*S*,7a*R*)-3-phenyltetrahydro-3*H*,5*H*-pyrrolo[1,2-*c*]oxazol-5-one (**24b**) (1.72 g, 8.48 mmol) in THF (100 mL) at −78 °C was added LDA (2 M in THF) (4.66 mL, 9.32 mmol), and the reaction mixture was allowed to warm to rt over 30 min. The resulting mixture was cooled to −78 °C, and iodomethane (0.53 mL, 8.48 mmol) was added dropwise. The reaction mixture was allowed to warm to rt, stirred for 4 h, and then quenched upon the addition of water (20 mL). The mixture was extracted with EtOAc (3 × 75 mL). The organic layers were combined and washed with sat. brine (50 mL), and the solvent was removed at reduced pressure. The resulting yellow oil was purified by flash column chromatography (Biotage 50 g Ultra, 12–100% EtOAc in cyclohexane) to yield the minor isomer **25b** as a yellow oil (263 mg, 1.21 mmol, 14%). δ_H_ (500 MHz, CDCl_3_): 7.48–7.42 (m, 2H), 7.39–7.26 (m, 3H), 6.31 (s, 1H), 4.20 (ddd, *J* = 7.6, 6.0, 1.4 Hz, 1H), 4.11–4.02 (m, 1H), 3.43–3.37 (m, 1H), 2.75–2.66 (m, 1H), 2.21–2.12 (m, 1H), 2.00–1.92 (m, 1H), 1.34 (dd, *J* = 7.4, 1.2 Hz, 3H).

##### Step 3: ((2*R*,4*S*)-1-Benzyl-4-methylpyrrolidin-2-yl)methanol (**26b**)

To a solution of (3*S*,6*S*,7a*R*)-6-methyl-3-phenyltetrahydro-3*H*,5*H*-pyrrolo[1,2-*c*]oxazol-5-one (**25b**) (263 mg, 1.21 mmol) in THF (25 mL) was added LiAlH_4_ (1 M in THF) (2.4 mL, 2.42 mmol), and the mixture was stirred at rt for 4 h. The reaction mixture was cooled to 0 °C and quenched with the dropwise addition of water (0.6 mL), aqueous NaOH (2 M) (1 mL), and water (2.7 mL). The resulting suspension was diluted with Et_2_O (10 mL) and stirred at rt for 30 min. Na_2_SO_4_ and Celite were added, and the slurry was filtered and washed with Et_2_O (100 mL). The filtrate was concentrated at reduced pressure to give **26b** as a colourless oil (256 mg, 1.21 mmol, >99%). δ_H_ (600 MHz, CDCl_3_): 7.39–7.26 (m, 5H), 3.96 (d, *J* = 12.9 Hz, 1H), 3.65 (dd, *J* = 10.7, 3.4 Hz, 1H), 3.43–3.36 (m, 2H), 3.08–3.02 (m, 1H), 2.88–2.83 (m, 1H), 2.20–2.12 (m, 1H), 2.05–1.94 (m, 2H), 1.60–1.53 (m, 1H), 0.98 (d, *J* = 6.6 Hz, 3H).

##### Step 4: (3*S*,5*S*)-1-Benzyl-5-methylpiperidin-3-ol (**27b**)

A solution of ((2*R*,4*S*)-1-benzyl-4-methylpyrrolidin-2-yl)methanol (**26b**) (263 mg, 1.28 mmol) in THF (15 mL) was cooled to −78 °C, and to this was added trifluoroacetic acid anhydride (214 μL, 1.54 mmol). The mixture was stirred at −78 °C for 45 min and then allowed to warm to rt and stirred for a further 45 min. The reaction mixture was cooled to −78 °C, triethylamine (0.71 mL, 5.12 mmol) was added, and the resulting solution was stirred at −78 °C for 15 min before heating at reflux for 16 h. The reaction mixture was cooled to rt, and aqueous NaOH solution (2 M) (2.11 mL, 4.23 mmol) was added, and the solution was stirred for 3 h. DCM (20 mL) was added, and the solution was washed with sat. NH_4_Cl solution (15 mL), sat. NaHCO_3_ solution (15 mL), and sat. brine (15 mL). The organic layer was dried over MgSO_4_, and the solvent was removed at reduced pressure. The resulting residue was purified by flash column chromatography (Biotage 50 g KP-sil, 20–80% EtOAc in cyclohexane) to give **27b** as a yellow oil (64 mg, 0.31 mmol, 22%). δ_H_ (500 MHz, CDCl_3_): 7.36–7.24 (m, 5H), 3.92–3.87 (m, 1H), 3.52 (s, 2H), 2.90–2.85 (m, 1H), 2.83–2.78 (m, 1H), 2.12 (dd, *J* = 11.3, 1.7 Hz, 1H), 2.05–1.96 (m, 1H), 1.88–1.82 (m, 1H), 1.63 (t, *J* = 10.9 Hz, 1H), 1.05 (ddd, *J* = 13.4, 12.1, 2.6 Hz, 1H), 0.86 (d, *J* = 6.6 Hz, 3H).

##### Step 5: (3*S*,5*S*)-5-Methylpiperidin-3-ol (**28b**)

To a solution of (3*S*,5*S*)-1-benzyl-5-methylpiperidin-3-ol (**28b**) (64 mg, 0.31 mmol) in ethanol (5 mL) under argon was added Pd/C (10 wt %; 33 mg, 0.031 mmol). The flask was evacuated and back-filled with hydrogen twice before being stirred at room temperature under a hydrogen balloon for 3 h. The reaction mixture was filtered through Celite (eluent methanol), and the filtrate was concentrated in vacuo to give the title compound (38 mg, 0.31 mmol, >99%) as a white solid, which was used without further purification. δ_H_ (500 MHz, CD_3_OD): δ 3.86 (t, *J* = 3.3 Hz, 1H), 2.97–2.90 (m, 1H), 2.85 (dt, *J* = 13.6, 2.9 Hz, 1H), 2.70 (dd, *J* = 13.5, 2.1 Hz, 1H), 2.22 (dd, *J* = 12.9, 10.6 Hz, 1H), 2.04–1.93 (m, 1H), 1.87–1.80 (m, 1H), 1.38–1.29 (m, 1H), 0.87 (d, *J* = 6.7 Hz, 3H).

##### Step 6: (*S*)-10-((5-chloro-2-((3*S*,5*S*)-3-hydroxy-5-methylpiperidin-1-yl)pyrimidin-4-yl)amino)-2-cyclopropyl-3,3-difluoro-7-methyl-1,2,3,4-tetrahydro-[1,4]oxazepino[2,3-*c*]quinolin-6(7*H*)-one (**13b**)

The method is the same as for **8**, using (3*S*,5*S*)-5-methylpiperidin-3-ol (**28b**). Purification by reverse-phase chromatography eluting from 30 to 100% methanol in water (both modified with 0.1% formic acid) was performed to give **13b** (3.9 mg, 0.0071 mmol, 22%). HRMS (ESI +ve): found 547.2020 expected 547.2030 for C_26_H_30_ClF_2_N_6_O_3_ [M + H]^+^; δ_H_ (600 MHz, CD_3_OD): 8.12 (d, *J* = 2.3 Hz, 1H), 7.94 (s, 1H), 7.91 (dd, *J* = 9.1, 2.3 Hz, 1H), 7.56 (d, *J* = 9.1 Hz, 1H), 4.56–4.38 (m, 2H), 4.32 (td, *J* = 14.7, 14.1, 3.7 Hz, 2H), 3.97 (s, 1H), 3.74 (s, 3H), 3.32–3.27 (m, 1H), 3.20–3.14 (m, 1H), 2.70 (dd, *J* = 12.9, 10.0 Hz, 1H), 2.10–2.00 (m, 1H), 1.89–1.81 (m, 1H), 1.50–1.37 (m, 2H), 0.91 (d, *J* = 6.7 Hz, 3H), 0.84–0.77 (m, 1H), 0.71–0.64 (m, 1H), 0.64–0.58 (m, 1H), 0.34 (s, 1H).

#### (*S*)-10-((5-chloro-2-((*R*)-3-hydroxypiperidin-1-yl)pyrimidin-4-yl)amino)-2-cyclopropyl-3,3-difluoro-7-methyl-1,2,3,4-tetrahydro-[1,4]oxazepino[2,3-*c*]quinolin-6(7*H*)-one (**14a**)

The method is the same as for **8**, using (*R*)-piperidin-3-ol hydrochloride. Purification by reverse-phase chromatography eluting from 40 to 90% methanol in water (both modified with 0.1% formic acid), followed by further purification using an SCX-2 column, was performed to give **14a** (3.1 mg, 0.0058 mmol, 22%). HRMS (ESI +ve): found 533.1880 expected 533.1874 for C_25_H_28_ClF_2_N_6_O_3_ [M + H]^+^; δ_H_ (600 MHz, CD_3_OD): 8.06 (d, *J* = 2.4 Hz, 1H), 7.98 (dd, *J* = 9.1, 2.3 Hz, 1H), 7.95 (s, 1H), 7.56 (d, *J* = 9.1 Hz, 1H), 4.58–4.36 (m, 2H), 4.31–4.20 (m, 1H), 4.11–4.00 (m, 1H), 3.73 (s, 3H), 3.66–3.55 (m, 1H), 3.38–3.26 (m, 1H), 3.21–3.14 (m, 1H), 3.09 (dd, *J* = 12.9, 8.3 Hz, 1H), 2.02–1.90 (m, 1H), 1.86–1.73 (m, 1H), 1.59–1.34 (m, 3H), 0.87–0.76 (m, 1H), 0.73–0.64 (m, 1H), 0.64–0.56 (m, 1H), 0.44–0.33 (m, 1H).

#### (*S*)-10-((5-chloro-2-((*S*)-3-hydroxypiperidin-1-yl)pyrimidin-4-yl)amino)-2-cyclopropyl-3,3-difluoro-7-methyl-1,2,3,4-tetrahydro-[1,4]oxazepino[2,3-*c*]quinolin-6(7*H*)-one (**14b**)

The method is the same as for **8**, using (*S*)-piperidin-3-ol hydrochloride. Purification by reverse-phase chromatography eluting from 40 to 90% methanol in water (both modified with 0.1% formic acid), followed by further purification using an SCX-2 column, was performed to give **14b** (2.7 mg, 0.0051 mmol, 14%). HRMS (ESI +ve): found 533.1882 expected 533.1874 for C_25_H_28_ClF_2_N_6_O_3_ [M + H]^+^; δ_H_ (600 MHz, CD_3_OD): 8.05 (d, *J* = 2.3 Hz, 1H), 7.97 (dd, *J* = 9.1, 2.3 Hz, 1H), 7.94 (s, 1H), 7.56 (d, *J* = 9.1 Hz, 1H), 4.54–4.35 (m, 2H), 4.28–4.20 (m, 1H), 4.11–4.02 (m, 1H), 3.72 (s, 3H), 3.67–3.59 (m, 1H), 3.39–3.26 (m, 1H), 3.20–3.12 (m, 1H), 3.08 (m, 1H), 2.03–1.93 (m, 1H), 1.83–1.74 (m, 1H), 1.57–1.37 (m, 3H), 0.86–0.75 (m, 1H), 0.72–0.65 (m, 1H), 0.65–0.58 (m, 1H), 0.42–0.32 (m, 1H).

#### (*S*)-10-((5-chloro-2-((*S*)-3-methylpiperidin-1-yl)pyrimidin-4-yl)amino)-2-cyclopropyl-3,3-difluoro-7-methyl-1,2,3,4-tetrahydro-[1,4]oxazepino[2,3-*c*]quinolin-6(7*H*)-one (**15a**)

The method is the same as for **8**, using (3*S*)-3-methylpiperidine hydrochloride. Purification by reverse-phase chromatography eluting from 40 to 90% methanol in water (both modified with 0.1% formic acid), followed by further purification using an SCX-2 column, was performed to give **15a** (4.1 mg, 0.0077 mmol, 27%). HRMS (ESI +ve): found 531.2076 expected 531.2081 for C_26_H_30_ClF_2_N_6_O_2_ [M + H]^+^; δ_H_ (600 MHz, CD_3_OD): 8.07 (d, *J* = 2.3 Hz, 1H), 7.96 (dd, *J* = 9.1, 2.3 Hz, 1H), 7.93 (s, 1H), 7.54 (d, *J* = 9.2 Hz, 1H), 4.59–4.29 (m, 4H), 3.73 (s, 3H), 3.39–3.27 (m, 1H), 2.83 (td, *J* = 12.7, 3.0 Hz, 1H), 2.51 (dd, *J* = 12.8, 10.6 Hz, 1H), 1.88–1.79 (m, 1H), 1.73–1.62 (m, 1H), 1.60–1.52 (m, 1H), 1.51–1.37 (m, 2H), 1.22–1.13 (m, 1H), 0.90 (d, *J* = 6.6 Hz, 3H), 0.83–0.76 (m, 1H), 0.71–0.64 (m, 1H), 0.65–0.58 (m, 1H), 0.41–0.31 (m, 1H).

#### (*S*)-10-((5-Chloro-2-((*R*)-3-methylpiperidin-1-yl)pyrimidin-4-yl)amino)-2-cyclopropyl-3,3-difluoro-7-methyl-1,2,3,4-tetrahydro-[1,4]oxazepino[2,3-*c*]quinolin-6(7*H*)-one (**15b**)

The method is the same as for **8**, using (3*R*)-3-methylpiperidine hydrochloride. Purification by reverse-phase chromatography eluting from 40 to 90% methanol in water (both modified with 0.1% formic acid), followed by further purification using an SCX-2 column, was performed to give **15b** (4.0 mg, 0.0075 mmol, 28%). HRMS (ESI +ve): found 531.208 4 expected 531.2081 for C_26_H_30_ClF_2_N_6_O_2_ [M + H]^+^; δ_H_ (600 MHz, CD_3_OD): 8.05 (d, *J* = 2.3 Hz, 1H), 7.98 (dd, *J* = 9.1, 2.3 Hz, 1H), 7.93 (s, 1H), 7.54 (d, *J* = 9.1 Hz, 1H), 4.53–4.35 (m, 5H), 3.72 (s, 3H), 2.84 (ddd, *J* = 13.1, 11.9, 3.0 Hz, 1H), 2.51 (dd, *J* = 13.0, 10.6 Hz, 1H), 1.87–1.78 (m, 1H), 1.72–1.65 (m, 1H), 1.62–1.37 (m, 3H), 1.22–1.12 (m, 1H), 0.90 (d, *J* = 6.7 Hz, 3H), 0.85–0.77 (m, 1H), 0.70–0.65 (m, 1H), 0.64–0.59 (m, 1H), 0.41–0.33 (m, 1H).

## Supporting Information Available

Molecular formula strings with the associated biochemical assay data and calculated properties (CSV)
Protein production, purification, and crystallography (BCL6 constructs used, methods for expression, purification, and crystallography, including data collection, processing, and refinement); small-molecule crystallography of intermediate **22b**; biological assay conditions (methods for TR-FRET, MSD, and cell proliferation assays); physicochemical and in vitro DMPK assay conditions (methods for NMR and HPLC solubility, log *D*, microsomal clearance, PAMPA, and protein binding); *in vivo* PK and PD experimental methods, including preparation of tumor xenografts; further analytical data for compound **1** (**CCT373566**) including LC–MS, chiral HPLC, and qNMR (XLSX)
Summary statistics and individual replicate values of TR-FRET and MSD assays; crystallographic data collection and refinement statistics; composition of solution formulation for **CCT373566** and **CCT369260**; and qNMR purity analysis for **CCT373566** (PDF)
X-ray structure of the BCL6 BTB domain with **CCT373566** highlighting key interactions between the protein and ligand; free mean mouse (BALB/c) blood concentrations (nM) of **CCT373566** and **CCT369260** after po dosing at 5 mg/kg; free mean blood concentration of compound **CCT373566** following a single 50 mg/kg po dose; PD effect of compound **CCT373566** on total plasma and tumor BCL6 levels following a single 50 mg/kg po dose; PD effect of compound **CCT373566** on total plasma and tumor BCL6 levels following 22 days of 50 mg/kg po BID dosing; chiral HPLC chromatogram of intermediate **22a**; X-ray crystal structure of intermediate **22b**; selectivity data for compound **1** (**CCT373566**); and PDB Validation report for 7QK0 (PDF)

## Accession Codes

Atomic coordinates and structure factors for the crystal structure of BCL6 with **CCT373566** can be accessed using PDB code 7QK0. Authors will release the atomic coordinates and experimental data upon article publication.

## Abbreviations

APCI: atmospheric pressure chemical ionization
BCL6: B-cell lymphoma 6 protein
BCOR: BCL-6 corepressor protein
BTB: Broad-complex, tramtrack, and bric a brac (domain), also known as POZ (poxvirus and zinc finger) domain
CL: clearance
CL_int_: intrinsic clearance
CL_u_: unbound clearance
c_max_: maximum (peak) concentration achieved after a single dose
DCM: dichloromethane
DC_50_: concentration of the compound at which 50% of the maximally observed protein degradation (D_max_) is achieved
DIPEA: *N,N*-diisopropylethylamine
DLBCL: diffuse large B-cell lymphoma
D_max_: maximal percentage degradation of protein achieved
EtOAc: ethyl acetate
GAPDH: glyceraldehyde 3-phosphate dehydrogenase
HAC: heavy atom count
MeCN: acetonitrile
MLM: mouse liver microsomes
MSD: meso scale discovery, an assay method similar to ELISA, using electrochemiluminescence as a detection technique
NCoR: nuclear receptor corepressor 1
PFP: pentafluorophenyl
PPI: protein–protein interaction
QToF: quadrupole time-of-flight
SCID: severe combined immunodeficient (mouse model)
SCX-2: SCX (strong cation exchange)-2 is a propylsulfonic acid-bonded sorbent
SMRT: silencing mediator for retinoid or thyroid hormone receptor (also known as nuclear receptor co-repressor 2 or NCoR2)
S_N_Ar: nucleophilic aromatic substitution
sol.: solubility
STR: short tandem repeat (analysis)
TR-FRET: time-resolved fluorescence energy transfer
