# Supplementary material for: Improved Binding Affinity and Pharmacokinetics Enable Sustained Degradation of BCL6 In Vivo
Source: J Med Chem. 2022 Jun 2;65(12):8191–207. doi: 10.1021/acs.jmedchem.1c02175 (PMC9234961; doi:10.1021/acs.jmedchem.1c02175)
Supplement: Supplementary file 3 — jm1c02175_si_003.pdf [file jm1c02175_si_003.pdf]

Supplementary information for:

Improved Binding Affinity and Pharmacokinetics Enables Sustained Degradation of BCL6 *In Vivo*

Rosemary Huckvale[†,‖], Alice C. Harnden[†,‖], Kwai-Ming J. Cheung[†], Olivier A. Pierrat[†], Rachel Talbot[†], Gary M. Box[†], Alan T. Henley[†], Alexis K. de Haven Brandon[†], Albert E. Hallsworth[†], Michael D. Bright[†], Hafize Aysin Akpinar[†], Daniel S. J, Miller[†], Dalia Tarantino[†], Sharon Gowan[†], Angela Hayes[†], Emma A. Gunnell[†,#], Alfie Brennan[†], Owen A. Davis[†], Louise D. Johnson[†], Selby de Klerk[†], Craig McAndrew[†], Yann-Vaï Le Bihan[†,#], Mirco Meniconi[†], Rosemary Burke[†], Vladimir Kirkin[†], Rob L. M. van Montfort[†,#], Florence I. Raynaud[†], Olivia W. Rossanese[†], Benjamin R. Bellenie[†,*], and Swen Hoelder[†,*]

Author Address

[†]Cancer Research UK Cancer Therapeutics Unit and [#]Division of Structural Biology, The Institute of Cancer Research, London SM2 5NG, U.K.

*E-mail: Benjamin.Bellenie@icr.ac.uk. Phone: +44 (0) 2087224602 (B.B.).

*E-mail: Swen.Hoelder@icr.ac.uk. Phone: +44 (0) 2087224353 (S.H.)

Contents

1. **Supplementary experimental: protein production, purification and crystallography** S-5
  1.1 **BCL6 constructs used for assays and crystallography** S-5
  1.2 **BCL6 expression** S-5
  1.3 **BCL6 purification** S-5
  1.4 **BCL6 crystallisation** S-6
  1.5 **Crystallographic data collection, processing and refinement** S-6
2. **Supplementary experimental: small molecule crystallography of 22b** S-7
2.1 **22b crystallisation, data collection, processing and refinement** S-7
3. **Supplementary experimental: biological assay conditions** S-8
  3.1 **TR-FRET assay** S-8
  3.2 **Mesoscale Discovery (MSD) Degradation Assay** S-9
  3.3 **Cell proliferation assay** S-10
4. **Supplementary experimental: physicochemical assays** S-11
  4.1 **NMR solubility assay** S-11
  4.2 **HPLC solubility assay** S-11
  4.3 **$logD_{7.4}$ assay** S-12
5. **Supplementary information: synthetic and analytical methods** S-14
5.1 **General Synthetic Information** S-14
5.2 **Analytical Methods** S-14
6. **Supplementary experimental: in vitro DMPK assays** S-16
  6.1 **Microsomal clearance assay** S-16
  6.2 **PAMPA permeability assay** S-16
  6.3 **Protein binding assay** S-17
7. **Supplementary experimental: in vivo PK and PD experiments** S-18
  7.1 ***In vivo* pharmacokinetic studies** S-18
  7.2 **Formulation of CCT373566 (12a) and CCT369260 (2)** S-19
  7.3 ***In vivo* pharmacodynamic (PK/PD) and efficacy studies** S-19
    7.3.1 Preparation of OCI-Ly1 Cells S-19
    7.3.2 Preparation of OCI-Ly1 xenograft model in mouse S-20
    7.3.3 Preparation of HT Cells S-20
    7.3.4 Preparation of HT xenograft model in mouse S-21
    7.3.5 Tissue sampling S-21
    7.3.6 *Tumour lysis and Wes method* S-21
8. **Analytical data for CCT373566 (12a)** S-23
  8.1 **LCMS method** S-23
  8.2 **HPLC and LCMS traces for CCT373566** S-23
  8.3 **qNMR method** S-24
  8.4 **qNMR analysis for CCT373566** S-25
9. **Supplementary Tables** S-26
Supplementary Table S1: S-26
Supplementary Table S2: S-27
Supplementary Table S3: S-28
Supplementary Table S4: S-29
Supplementary Table S5: S-29
10. **Supplementary Figures** S-30
Supplementary Figure S1: S-30

Supplementary Figure S2: S-30
Supplementary Figure S3: S-31
Supplementary Figure S4: S-32
Supplementary Figure S5: S-33
Supplementary Figure S6: S-34
Supplementary Figure S7: S-34
11. References for supplementary information S-35

1. Supplementary experimental: protein production, purification and crystallography

1.1 BCL6 constructs used for assays and crystallography

A first construct of BCL6 BTB domain, which we will refer to as Trx-6His-HRV3C-BCL6, was obtained by sub-cloning the sequence coding for residues 5-129 of human BCL6, corresponding to its BTB domain, into a pET48b vector with N-terminal Thioredoxin and 6-Histidine tags, followed by a HRV-3C protease cleavage site. For the TR-FRET assay, the Trx-6His-HRV3C-BCL6 protein construct was used without cleaving the tag, as a 6His was needed to bind to the anti-6His-Terbium antibody.

For crystallography with compound **12a** (**CCT373566**), the construct described above was modified to introduce a Flag Tag and a TEV cleavage site between the HRV3C and BCL6 sequences. This construct will be referred to as Flag-TEV-BCL6.

1.2 BCL6 expression

For both plasmid constructs described above, transformed BL21-AI E. coli cells were grown in LB media supplemented with 50 mg/L kanamycin at 37 ^{o}C until an $OD_{600\ nm}$ of 0.6 was reached. Protein expression was then induced by addition of 0.2 mM IPTG and 0.2 % Arabinose. Expression was carried out at 18 ^{o}C for 18 hours. Cells were harvested by centrifugation (5500 g for 30 minutes at 4 ^{o}C) and stored at -80 ^{o}C.

1.3 BCL6 purification

Cells were re-suspended in a buffer composed of 20 mM Tris pH 8, 250 mM NaCl, 1 mM $MgCl_2$, 0.5 mM TCEP and 5 % glycerol, 1x cOmplete^{TM} ULTRA protease inhibitors and 12.5 U/ml Benzonaze. Cells were lysed by sonication followed by centrifugation at 21,000 g for 45 minutes at 4 ^{o}C. The supernatant was loaded onto a HisTrap FF column followed by on-column cleavage of the Trx-6His-HRV3C tag by addition of 2 mg of HRV-3C protease. The cleaved Flag-TEV-BCL6 5-129 was then eluted and purified further by

ResourceQ (for Flag-TEV-BCL6 construct only) and gel filtration using a HiLoad 26/60 Superdex75 column in a buffer containing 20 mM HEPES pH 7.5, 250 mM NaCl, 1 mM TCEP and 5 % glycerol. The final protein was assessed for purity and molar mass by SDS-PAGE and high-resolution mass spectrometry, respectively.

For the uncleaved Trx-6His-HRV3C-BCL6 protein construct to be used in TR-FRET, the protein was directly eluted from the HisTrap FF column without HRV-3C treatment, and submitted to Superdex75 gel filtration as described above.

1.4 BCL6 crystallisation

The purified Flag-TEV-BCL6 5-129 was crystallised without any peptide, the supplementary Flag-TEV tag of this construct replacing the WVIP peptide in the crystal packing. The purified Flag-TEV-BCL6 5-129 was concentrated to a final protein concentration of 11 mg/mL using a centrifugal concentrator with a 10 kDa molecular weight cut-off. Crystals were grown at 18 ^{o}C in hanging drops composed of 1.5 µL of the Flag-TEV-BCL6 complex plus 1.5 µL of a crystallisation solution consisting of 0.1 M Tris pH 7.5 and 0.80 M Na/K Tartrate, against 300 µL of crystallisation solution. Crystals typically grew in 2 days, and compound **CCT373566** was soaked into crystals by addition of 0.5 µL (dissolved in DMSO to a final concentration of 50 mM) directly to crystallisation drops, followed by 48 hours incubation. Crystals were then cryo-protected in a solution composed of the crystallisation reagent supplemented with 30 % ethylene glycol and cryo-cooled in liquid nitrogen.

1.5 Crystallographic data collection, processing and refinement

X-ray data were collected at Diamond Light Source, Harwell campus, Oxfordshire, UK, on beamlines I03. The chosen crystal belonged to the space group P 6_1 2 2 and diffracted to 1.96 Å resolution. Dataset was integrated with XDS[1] and scaled and merged with AIMLESS[2]. Structure was solved by molecular

replacement using PHASER[3] with a publicly available BCL6 structure[4] (PDB code 3BIM) with ligand and water molecules removed used as molecular replacement model. The protein/ligand structure was manually corrected and rebuilt in COOT[5] and refined with BUSTER[6] in iterative cycles. Ligand restraints were generated with GRADE[7] and MOGUL[8]. The quality of the structure was assessed with MOLPROBITY[9]. The data collection and refinement statistics are presented in Table S3.

2. Supplementary experimental: small molecule crystallography of 22b

2.1 22b crystallisation, data collection, processing and refinement

Intermediate **22b** was isolated as the later eluting isomer from a racemic mixture of *rac*-1-benzyl-5-methylpiperidin-3-ol hydrogen chloride by SFC (Lux A1 (30 mm x 250 mm, 5 μm), 10:90 MeOH:CO_2 (0.2% v/v DEA; flow rate 100 mlmin^{-1}). Crystals suitable for X-ray diffraction were grown from DCM and diethyl ether and the data was collected at the EPSRC National Crystallography Service.[10]

3. <u>Supplementary experimental: biological assay conditions</u>

Cell lines were supplied by the German Collection of Microorganisms and Cell Cultures (DSMZ). Cell lines were authenticated by STR profiling using a GenePrint® 10 kit (Promega, Southampton, UK) and a 3730xl DNA analyser (Applied Biosystems, Warrington, UK). All STR profiles were >80% match (using ATCC or DSMZ matching algorithms) with the respective reference profile. Cells were routinely screened for *Mycoplasma*, using an in-house PCR-based assay (Universal Mycoplasma Detection Kit (30-1012K, ATCC, Manassas, VA, USA).

3.1 TR-FRET assay

Assays were performed in a 384-well black Proxiplate (Perkin Elmer) containing 1 nM Trx-6xHis-BCL6 (in house-produced, human BCL6 BTB domain covering amino-acid sequence 5-129), 300 nM BCOR-AF633 peptide (RSEIISTAPSSWVVPGP-Cys-AlexaFluor 633-amide, Cambridge Research Biochemical) and 0.5 nM anti-6xHis-Terbium cryptate (CisBio Bioassays, France), in assay buffer (25 mM Hepes pH8, 100 mM NaCl, 0.05% Tween20, 0.5 mM TCEP, 0.05% bovine serum albumin). Test compounds in DMSO or DMSO alone were added to the wells using an ECHO550 acoustic dispenser (Labcyte Inc) to give the appropriate test concentration in 0.7% v/v DMSO final. After 2 hours incubation at room temperature the plate was read on an Envision plate reader (Perkin Elmer) with 337 nm laser excitation, a first emission filter APC 665 nm and a second emission filter Europium 615 nm, or alternatively on a Pherastar FSX (BMG Labtech) plate reader equipped with 337 nm laser excitation filter, a first emission filter at 620 nm and a second emission filter at 665 nm. The % inhibition at each concentration was calculated by normalising FRET ratio to the appropriate high (DMSO with all reagents) and low (DMSO without BCL6) controls. IC_{50} values were determined using GraphPad Prism 6.0 or Dotmatics (Bishops Stortford, UK) software by fitting the normalised data to a sigmoidal four-parameter logistic fit equation.

3.2 Mesoscale Discovery (MSD) Degradation Assay

An MSD assay was developed for screening of compounds to determine the degradation of endogenous BCL6 in OCI-Ly1 cells. Briefly, test compounds in DMSO or DMSO alone to a total of 1332.5 nL/well were dispensed to a 96-well Nunc™ Edge 2 plate (ThermoFisher Scientific, 267544) using an Echo® 550 acoustic dispenser (Labcyte Inc) to give the appropriate test concentration in 0.67% v/v DMSO final. OCI-Ly1 passaged in Iscove's Modified Dulbecco's Medium (IMDM, ThermoFisher Scientific, 12440053) supplemented with 10% heat-inactivated fetal bovine serum (Sera Plus, PAN Biotech, P30-3702) were added to the compound/DMSO at a density of 2.5 x 10^5 cells/mL in 200 µL media/ well. After 2 hours incubation at 37 °C + 5% CO_2 the plate was centrifuged at 300 x g for 5 minutes. Using a BioTek 405TS plate washer for all wash and aspiration steps, media was removed and cells were rinsed with PBS, centrifuged as before and PBS removed. Cells were lysed in 50 µL ice-cold lysis buffer at pH 7.4 containing 50 mM Tris-HCl, 150 mM NaCl, 1 % Triton X-100 (v/v), 1 mM PMSF, 1 mM activated sodium vanadate, 1 mM EDTA, phosphatase inhibitors 2 and 3 (1:50 dilution) and protease inhibitor cocktail (1:100 dilution). The plate was shaken briefly and incubated on ice for 5-10 minutes before being frozen overnight at -20 °C or continuing the assay. A 96-well MSD standard bind plate (MSD, L15XA-3) which was coated overnight with 1 µg/mL anti-human BCL6 goat antibody in PBS (R&D Systems, AF5046) was blocked in 3% BSA in TBS + 0.1% Tween-20 for 1 hour with shaking. Wells were emptied thoroughly before 40 µL/well of lysate was transferred from the cell plate. The MSD plate was incubated at RT for 1 hour with shaking then washed three times with TBS + 0.1 % Tween-20. Antibodies were prepared in TBS + 0.1% Tween-20 + 1% BSA. Anti-human BCL6 rabbit antibody (Cell Signalling Technology, 14895S) was added at 1:100 dilution at 25 µL/well. The plate was incubated and washed as before. MSD Sulfo-tag anti-rabbit detection antibody (MSD, R32AB-1) at 1:1000 dilution at 25 µL/well was added. The plate was incubated and washed as before and MSD Gold read buffer (MSD R92TG) at 1:1 dilution with water at 150 µL/well was added. The plate was read within 10 minutes on the MSD Quickplex reader. The % inhibition at each

concentration was calculated by normalising the electrochemiluminescent signal to the appropriate high (DMSO) and low (**CCT369260** at 2 µM) controls. DC_{50} values (compound concentration at which 50% of endogenous BCL6 protein is degraded) were determined using GraphPad Prism 6.0 or Dotmatics (Bishops Stortford, UK) software by fitting the normalised data to a sigmoidal four-parameter logistic fit equation.

3.3 Cell proliferation assay

Cells were seeded in 96-well culture plates at a density of 2500 cells/well in RPMI-1640 medium (Sigma-Aldrich) supplemented with 10% FBS (Gibco). Compounds were initially dispensed into 96-well U-bottom plates using an Echo 550 acoustic dispenser (Labcyte Inc.), then diluted in RPMI-1640 medium and transferred onto the cells. Cells were treated with 8 compound concentrations in duplicate, ranging from 1.07 nM to 10 µM, in a final DMSO concentration of 0.1% and final volume of 100 µl. Cells were incubated with compound for 14 days, with medium changes at days 3, 7 and 10 carried out as follows: fresh 96-well cell culture plates were prepared containing 100 µl medium plus compound at the assay concentrations (white plates were used on day 10 to optimise luminescence measurement). Assay plates containing cells were vortexed to mix and cell density in one control well was counted using a Coulter Z2 cell counter (Beckman Coulter). The volume of medium containing 2500 cells in the control well was calculated and this volume of cells was transferred from every well of the assay plates to the corresponding well of the fresh plates containing compound. After 14 days, CellTiter Glo reagent (Promega) was added to the medium in each well of the assay plate at a ratio of 1:2, mixed on a plate shaker, then incubated at room temperature for 10 minutes. Luminescence was measured using an Envision plate reader (Perkin Elmer) and the relative luminescence at each compound concentration, compared to DMSO alone, was calculated. GI50 were determined using a 4-parameter curve fit in Dotmatics (Bishops Stortford, UK).

4. Supplementary experimental: physicochemical assays

4.1 NMR solubility assay

9 µL of 10 mM DMSO stock solution was pipetted into one well of a 384 deepwell plate (Greiner, part-no. 781270), then 171 µL of HEPES buffer (20 mM HEPES [Sigma Aldrich, cat-no. H3375-250G], 150 mM NaCl, 0.5 mM TCEP, 10% D_2O) was pipetted into the same well and mixed by up-down pipetting 3 times to create a 0.5 mM solution or suspension containing 5% DMSO. It was then separated by centrifugation (1000 rpm for 1 min, Eppendorf 5810C). The plate was then sealed and incubated at room temperature for 20 hours, without shaking. The plate was centrifuged again for 1 minute at 1000 rpm on Eppendorf 5810R before 165 µL of the supernatant was transferred to a 3 mm NMR tube (Bruker, Part No. Z112272) using liquid handler SamplePro Tube SJ S (Bruker). The concentration of the solubilized compound in solubility sample is measured by quantitative ^{1}H-NMR using a single external standard (200 µM caffeine (Sigma, C1778) in PBS (pH 7.4) with 1% DMSO-d6).

The detail of the NMR method is as following: NMR data was collected on a Bruker Avance Neo 600 spectrometer equipped with a 5 mm TCI-CryoProbe. The ^{1}H spectrum was referenced to the internal deuterated solvent. The operating frequency for ^{1}H was 600 MHz. All NMR data were acquired at the temperature of 298 K. All data were acquired and processed using Bruker Topspin 4.0. The quantitative ^{1}H-NMR spectrum was acquired using a Bruker standard 1D lc1pngppsf2 pulse sequence with 32 scans. The sweep width was 6.2 ppm with O1P set to 8.8 ppm, and the FID contained 16k time-domain data points. Relaxation delay was set to 20 sec. Water signal was suppressed. [11]

4.2 HPLC solubility assay

10 µL of 10 mM DMSO stock solution was pipetted into a micro centrifuge tube (1.5 mL, Sarstedt part-no. 72.690.001) containing 990 µL of PBS buffer (pH 7.4, Sigma Aldrich, cat-no. 79382) and mixed for 5

seconds on vortex mixer (Grant-bio vortex mixer) to create 100 µM solution with 1% DMSO. Following shaking of the suspension on a Bohdan Shaker at 500 rpm for 2 hours at room temperature (20 °C), it was separated by centrifugation (14000 rpm for 15 min, Eppendorf 5415C). 200 µL of the supernatant was transferred to a 2 mL Agilent vial containing 50 µL of DMSO (Sigma Aldrich, cat-no. 41640-100ML) and mixed for 5 seconds to avoid precipitation from the saturated solution.

The concentration of the solubilized compound in solubility sample is measured by HPLC with UV detection using an external standard which was prepared by pipetting 10 µL of the same batch of compound DMSO stock used in solubility sample preparation to 990 µL of DMSO.

The detail of the HPLC method is as following: chromatographic separation at 30°C is carried out over a 5 minute gradient elution method from 90:10 to 10:90 water:methanol (both modified with 0.1% formic acid) at a flow rate of 1.5 mL/min. Calibration curve is prepared by injecting 0.5, 2.5, and 5 µL of compound external standard. Compound solubility value is obtained by injecting 6.25 and 62.5 µL of compound solubility sample.

4.3 $logD_{7.4}$ assay

Calibration, validation and in-house compounds were prepared at 1mM in 10% DMSO / 90% Trizma solution (100mM Trizma in 75/25 methanol/water). 3 µL standard injections (with needle wash) of all calibration, validation and in-house samples were made onto a Phenomenex Luna C8 column (3µm, 100 x 4.6 mm, 100A, Phenomenex, Torrence, USA). Chromatographic separation at 30°C was carried out using a 1260 Series HPLC (Agilent, Santa Clara, USA) over a 5 minute gradient elution from 95:5 to 0:100 aqueous (20mM Trizma in octanol-saturated water) and organic (acetonitrile + 0.25% v/v octanol) at a flow rate of 2 mL/min. The gradient was held at 0:100 water:organic for 0.8 minutes, then returned to the starting conditions of 95:5 water:organic for 0.2 minutes. The column was re-equilibrated for 5 minutes at the

starting conditions prior to the next injection. UV-Vis spectra were acquired at 254 nm, 280 nm and 220 nm on a 1260 Series diode array detector (Agilent, Santa Clara, USA). Raw data was processed using Agilent Chemstation Rev.C.01.04.

5. Supplementary information: synthetic and analytical methods

5.1 General Synthetic Information

All anhydrous solvents and reagents were obtained from commercial suppliers and used without further purification. Evaporation of solvent was carried out using a rotary evaporator under reduced pressure at a bath temperature of up to 60 °C. Flash column chromatography was carried out using a Biotage purification system using prepacked SNAP KP-Sil cartridges. Reverse phase column chromatography was carried out using a Biotage purification system using Biotage SNAP Ultra C-18 12g and 30g columns as required. HPLC separations were carried out on an Agilent 6120 MS-Prep LC using an ACE 5 C18-PFP 250 x 21.2 mm column using a 15 min gradient of water:methanol (both modified with 0.1% formic acid).. Microwave-assisted reactions were carried out using a Biotage Initiator microwave system. Final compounds were purified to ≥ 95% purity.

5.2 Analytical Methods

NMR spectra were recorded on a Bruker AMX 500 (500 MHz) spectrometer, or on a Bruker Avance NEO 600 (600 MHz) spectrometer equipped with 5 mm TCI Cryoprobe. NMR data is presented in the form of chemical shift δ (multiplicity, coupling constants, integration) for major diagnostic protons, given in parts per million (ppm) relative to tetramethylsilane (TMS), referenced to the internal deuterated solvent.

LCMS/HRMS analysis was performed according to one of four methods as described in the experimental text. Where **2min; ToF** is shown, LC/MS and HRMS analysis was performed on an Agilent 1200 series HPLC and diode array detector coupled to a 6210 time of flight mass spectrometer with dual multimode APCI/ESI source. Analytical separation was carried out at 40°C on a Merck Chromolith Flash column (RP-18e, 25 x 2 mm) using a flow rate of 1.5 mL/min in a 2 minute gradient elution with detection at 254 nm. The mobile phase was a mixture of methanol (solvent A) and water (solvent B), both containing formic acid at 0.1%. Gradient elution was as follows: 5:95 (A/B) to 100:0 (A/B) over 1.25 min, 100:0 (A/B) for 0.5 min, and then reversion back to 5:95 (A/B) over 0.05 min, finally 5:95 (A/B) for 0.2 min. Where **4min; ToF** is shown, the method is the same except at 30°C and using a flow rate of 0.75 mL/min in a 4 minute gradient elution as follows: 5:95 (A/B) to 100:0 (A/B) over 2.5 min, 100:0 (A/B) for 1 min, and then reversion back to 5:95 (A/B) over 0.1 min, finally 5:95 (A/B) for 0.4 min. Where **2min; ESI** is shown, LC/MS and HRMS analysis was performed on a Waters Acquity UPLC and diode array detector coupled to a Waters G2 QToF mass spectrometer fitted with a multimode ESI/APCI source. Analytical separation was carried out at 30°C on a Phenomenex Kinetex C18 column (30 x 2.1 mm, 2.6u, 100A) using a flow rate of

0.5 mL/min in a 2 minute gradient elution with detection at 254 nm. The mobile phase was a mixture of methanol (solvent A) and water (solvent B), both containing formic acid at 0.1%. Gradient elution was as follows: 10:90 (A/B) to 90:10 (A/B) over 1.25 min, 90:10 (A/B) for 0.5 min, and then reversion back to 10:90 (A/B) over 0.15 min, finally 10:90 (A/B) for 0.1 min. Where **4min; ESI** is shown, the method is the same except using a flow rate of 0.3 mL/min in a 4 minute gradient elution as follows: 10:90 (A/B) to 90:10 (A/B) over 3 min, 90:10 (A/B) for 0.5 min, and then reversion back to 10:90 (A/B) over 0.3 min, finally 10:90 (A/B) for 0.2 min.

6. Supplementary experimental: in vitro DMPK assays

6.1 Microsomal clearance assay

Microsomal clearance was determined in female CD1 mice, female Sprague–Dawley rats, and mixed gender human liver microsomes obtained from BioIVT (Peterborough, U.K.) following incubation of 1 μM compound at 37 °C in 0.5 mg/mL microsomal protein, 3 mmol/L $MgCl_2$, 1 mmol/L NADPH, 2.5 mmol/L and 10 mmol/L phosphate buffer (pH 7.4) (all purchased from Sigma-Aldrich, Gillingham, U.K). Reactions were started by addition of the cofactors following 10 min preincubation of microsomes with test compound and were terminated at −1, 0, 5, 10, 15, and 30 min with three volumes of ice-cold methanol containing internal standard. Samples were centrifuged at 2800*g* for 30 min at 4 °C and the supernatants analyzed. Control incubations were prepared as above with omission of cofactors. Compound measurements were performed by LCMS on an Agilent quadrupole time-of-flight instrument (Agilent 6510) following separation with a 3 min gradient of 0.1% formic acid in methanol on a Kinetex C18, 50 × 2.1 mm 2.6 μm UPLC column (Phenomenex, Macclesfield, UK).

6.2 PAMPA permeability assay

Passive diffusion permeability was measured using a Parallel Artificial Membrane Permeability Assay (PAMPA). The assay used an artificial membrane consisting of 2% phosphatidyl choline in dodecane (Sigma Aldrich, Dorset, UK). The donor plate was a MultiScreen-IP Plate with 0.45 μm hydrophobe Immobilon-P Membrane (Millipore, UK) and the acceptor plate was a MultiScreen 96-well Transport Receiver Plate (Millipore, UK). The permeability of test compound (10 μM) was measured at pH 7.4 in phosphate buffered saline (PBS, Sigma Aldrich, Dorset, UK) containing 1% Bovine Serum Albumin (Sigma Aldrich, Dorset, UK) following a 16 hour incubation at 30 °C. After transfer and centrifugation, sample supernatants were diluted and analysed using a Waters (Milford, MA, US) TQ-S LC-MS/MS system.

Permeability values (cm/s) were calculated using the following equation:

$$P_{app} = C \times -\ln\left(1 - \frac{[\text{drug}_{acceptor}]}{[\text{drug}_{equilibrium}]}\right) \quad \text{where} \quad C = \frac{V_D \times V_A}{(V_D + V_A) \times \text{area} \times \text{time}}$$

V_D = volume of donor
V_A = volume of acceptor
Area = surface area of the membrane x porosity

6.3 Protein binding assay

Protein binding in mouse plasma (Charles River, Wilmington, MA, USA) and in Iscove's Modified Dulbecco's Medium (IMDM, Gibco, Life technologies Ltd, Paisley, UK) + 10% FBS was measured using Rapid Equilibrium Dialysis (RED, Thermo Fisher Scientific, Loughborough, UK). Test compound in DMSO was spiked into either mouse plasma or cell culture media containing protein as appropriate resulting in a concentration of 5 µM for dialysis, containing 1 % DMSO. 300 µL of spiked matrix was added to the donor side of the RED plate and 500 µL of dialysate (either 100 mM phosphate buffer or protein free media for plasma or media protein binding respectively) was added to the receiver well. Dialysis was performed by shaking for 4 h at 37 °C. After dialysis, samples were matrix matched followed by protein precipitation with acetonitrile containing internal standard. Samples were mixed, centrifuged and supernatant was taken for analysis by ESI-LCMS/MS on a Waters (Milford, MA, USA) Xevo TQ-S following gadient separation with 0.1% formic acid in water and acetonitrile on a Phenomenex (Macclesfield, UK) Kinetex C18 UPLC column (50 × 2.1 mm, 2.6 µM).

The fraction unbound (f_u) was calculated as follows:

$f_u' = \frac{PAR\ receiver}{PAR\ donor}$ where PAR = Peak Area Ratio of Analyte/Internal Standard.

$$f_u = 1/\left(1 + \left(\frac{1}{fu'} - 1\right)\right)$$

7. Supplementary experimental: in vivo PK and PD experiments

All procedures were in accordance with UK Home Office regulations under the Animals (Scientific Procedures) Act 1986, approved by The Institute of Cancer Research's Ethics Committee and in accordance with published guidelines.[12]

7.1 *In vivo* pharmacokinetic studies

Animals were adapted to laboratory conditions for at least 1 week prior to dosing and were allowed food and water *ad libitum*. Compounds were administered iv or po (mouse, 0.1 mL/10 g in 10% DMSO, 5% tween 20 in saline); blood samples were collected in heparinised capillaries from the tail vein at 7 time points over the 6 h post dose and frozen on collection together with a standard curve and quality controls spiked in control blood. Samples were reconstituted in a water:MeOH mixture containing internal standard as previously described (Roberts et al, 2016). Following centrifugation, extracts were analyzed by multiple reaction monitoring of precursor and product ions by ESI-LCMS/MS on either a Waters (Milford, MA, USA) Xevo TQ-S or Sciex (Framingham, MA, USA) QTrap6500 following gradient separation with 0.1% formic acid and methanol on a Phenomenex (Macclesfield, UK) Kinetex C18 UPLC column (50 × 2.1 mm, 2.6 µM). Quantitation was carried out with an external calibration. Quality controls were included and were within 20% of nominal concentration.

Pharmacokinetic parameters were derived from noncompartmental analysis using Phoenix Pharsight Non compartmental analysis (model 200 and 201) version 6.3.

7.2 Formulation of CCT373566 (12a) and CCT369260 (2)

A solution formulation suitable for higher concentrations (> 5 mg/mL) of both **CCT369260** and **CCT373566** was developed by SEDA as described in Table S4.

CCT373566 or **CCT369260** was dissolved in a pre-determined volume of DMSO (Sigma-Aldrich, UK) and to this was added a pre-determined volume of Kolliphor HS15 (Sigma-Aldrich, UK, 42966) at 40 °C. The solution was briefly vortexed before addition of a pre-determined volume of PEG400 (Sigma-Aldrich, UK, 8.07485) at 40 °C. After a brief vortex a pre-determined volume of aqueous HPMC (1.25%, viscosity 40-60cp grade, Sigma-Aldrich, UK, H8384) was added. The formulation was vortexed and then sonicated at 40 ^{o}C for ~45-60 mins. Complete vehicle was prepared in the same manner with no compound added. The formulation was stored at room temperature for 3-4 days maximum. Both compound and vehicle solutions were delivered orally (0.2 mL per 20 g mouse) using a gavage needle - mice were individually weighed and dosing volumes adjusted accordingly.

7.3 *In vivo* pharmacodynamic (PK/PD) and efficacy studies

7.3.1 Preparation of OCI-Ly1 Cells

The OCI-Ly1 cell line was supplied by the American Type Culture Collection (ATCC) and authenticated by standard tandem repeat (STR) profiling and screened for mycoplasma using an in-house PCR-based assay. B cell lymphoma cells, OCI-Ly1 were grown in Iscove's Modified Dulbecco's Medium (IMDM) (#12440, ThermoFisher Scientific, UK) supplemented with 15% fetal bovine serum (Sera Plus, #P30-3702, PAN Biotech UK Limited). Cells were maintained at a density between 0.5-2 x 10^6 cells/mL and near-saturated cultures were subcultured 1:4 every 3-4 days.

OCI-Ly1 B-cell lymphoma cells were grown in Iscove's Modified Dulbecco's Medium (IMDM) (#12440, ThermoFisher Scientific, UK) supplemented with 15% fetal bovine serum (Sera Plus, #P30-3702, PAN

Biotech UK Limited). Cells were maintained at a density between 0.5-2 x 10^6 cells/mL and near-saturated cultures were subcultured 1:4 every 3-4 days.

7.3.2 Preparation of OCI-Ly1 xenograft model in mouse

Cells were prepared for injection at a final concentration of 7.5 x 10^7, using serum–free IMDM and an equal volume of Matrigel®, (#354234, Corning® B.V., Netherlands) both chilled to 4°C. Cells were delivered to female SCID mice (NOD.CB17-*Prkdc*^{scid}/J, Charles River, UK) at 1.5 10^7 per 200uL, subcutaneously, single site.

Twenty days post injection 24 tumour-bearing mice (between 0.4 and 0.6 cm^3) were selected for the study and grouped as follows:

1. **CCT373566** 50 mg/kg per os (n=9)

   Mice were then grouped n=3 for the following time points: 12, 16 & 24 hours

2. Vehicle per os (n=9)

   Mice were then grouped n=3 for the following time points: 12, 16 & 24 hours

Compounds and vehicle were administered po (formulated as described in Supplementary experimental 7.2); group one received a single treatment of **CCT373566** (50 mg/kg) orally and group two received a single treatment of vehicle.

7.3.3 Preparation of HT Cells

The HT cell line was supplied by the German Collection of Microorganisms and Cell Cultures (DSMZ) and authenticated by standard tandem repeat (STR) profiling and screened for mycoplasma using an in-house PCR-based assay. HT B-cell lymphoma cells were grown in RPMI-1640 Medium (#72400), ThermoFisher Scientific, UK) supplemented with 10% fetal bovine serum (Sera Plus, #P30-3702, PAN Biotech UK Limited).

Cells were maintained at a density between 0.5-2 x 10^6 cells/mL and near-saturated cultures were subcultured 1:4 every 3-4 days.

7.3.4 *Preparation of HT xenograft model in mouse*

Cells were prepared for injection at a final concentration of 5 x10^7 cells/mL, using serum–free RMPI-1640 and an equal volume of Matrigel®, (#354234, Corning® B.V., Netherlands) both chilled to 4°C. Cells were delivered to female SCID mice (NOD.CB17-*Prkdc*^{scid}/J, Charles River, UK) at 10^7 per 200uL, subcutaneously, single site.

Twenty days post injection 20 tumour-bearing mice (between 0.4 and 0.6 cm^3) were selected for the study and grouped as follows:

1. **CCT373566** 50 mg/kg per os (n=10)
2. Vehicle per os (n=10)

Compounds and vehicle were administered po (formulated as described in Supplementary experimental 7.2); group one received **CCT373566** 50 mg/kg orally twice per day, and group two received vehicle twice per day. Animals were dosed at 12 h intervals and were treated for 22 days.

7.3.5 *Tissue sampling*

Blood (~1 mL) was taken from anaesthetized mice, via intracardiac puncture, using a 27 gauge needle and a syringe pre-coated with heparin (200 Units/mL), The heparinized blood was microcentrifuged for 2 minutes and ~200 uL of plasma was aspirated to a labelled tube, frozen on dry-ice and dedicated to pharmacokinetic analysis (PK). Each tumour was excised, divided into two halves, weighed and then snap frozen in liquid nitrogen, dedicating one half to PK analysis and the other half to pharmacodynamic analysis (PD). All frozen material was permanently stored in a -80°C freezer.

7.3.6 *Tumour lysis and Wes method*

Tumours were lysed in lysis buffer (50 mM Tris-HCl, pH 7.4, 150 mM NaCl, 1% Triton X-100 (v/v), 1 mM PMSF, 1 mM activated sodium vanandate, 1 mM EDTA, phosphatase inhibitors 2 and 3 (1:50m dilution) and protease cocktail (1:100 dilution)) using reinforced homogenizing tubes containing metal beads using a Precellys 24 with cryolys cooling at 6000 rpm, 2 × 20 s (Stretton Scientific). Lysates were incubated on ice for 15 minutes, spun at 14000 rpm at 4 °C for 10 minutes and supernatants collected and aliquoted. Samples were stored at -80 ^{o}C until use. Protein concentration was determined by diluting 1:10 with lysis buffer and measuring on Direct Detect® spectrometer according to manufacturer's instructions.

Protein lysates were diluted to 0.0875 mg/ml with 0.1x sample buffer and run on Wes 12-230 kDa kit according to manufacturer's instructions (ProteinSimple) with multiplexing BCL6 (CST14895 at 1:150 dilution) and GAPDH (CST 2118 at 1:50 dilution). Data was analysed using Compass for SW software. BCL6 data was normalised to GAPDH and transferred to GraphPad Prism 7.

8. <u>Analytical data for CCT373566 (12a)</u>

8.1 LCMS method

LC/MS and HRMS analysis for compounds shown below was performed on an Agilent 1200 series HPLC and diode array detector coupled to a 6210 time of flight mass spectrometer with dual multimode APCI/ESI source. Analytical separation was carried out at 30 ^{o}C on a Merck Chromolith Flash column (RP-18e, 25 x 2 mm) using a flow rate of 0.75 mL/min in a 4 minute gradient elution with detection at 254 nm. The mobile phase was a mixture of methanol (solvent A) and water (solvent B), both containing formic acid at 0.1%. Gradient elution was as follows: 5:95 (A/B) to 100:0 (A/B) over 2.5 min, 100:0 (A/B) for 1 min, and then reversion back to 5:95 (A/B) over 0.1 min, finally 5:95 (A/B) for 0.4 min.

8.2 HPLC and LCMS traces for CCT373566

*High resolution LCMS trace of **CCT373566***

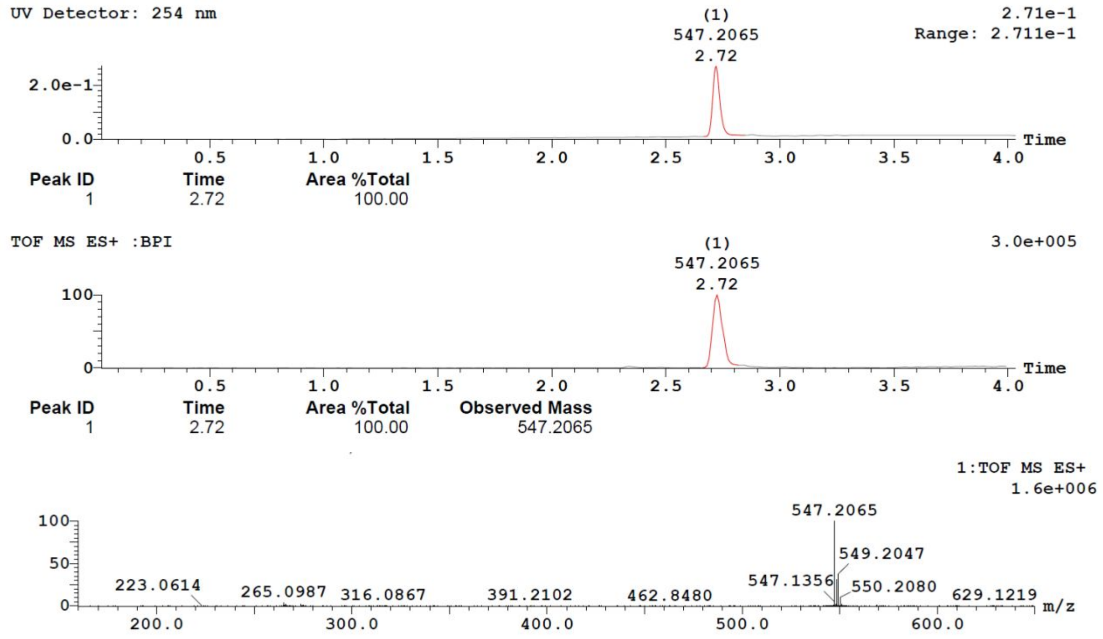

Chiral HPLC analysis of CCT373566

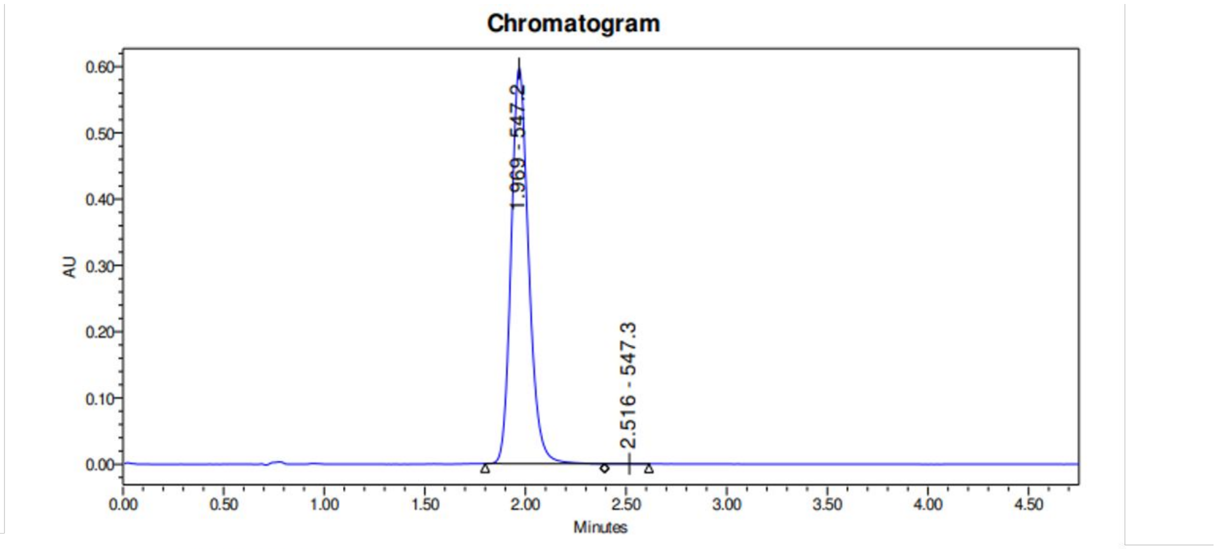

Peak Results

	Retention Time (min)	Area (µV*sec)	% Area	Width @ 50%
1	1.97	3607603	100.0	0.09138
2	2.52	1151	0.0	

**Column Details**	AMS (4.6 mm x 250 mm, 5 um)
**Column Temperature**	40 °C
**Flow Rate**	4 mL/min
**Detector Wavelength**	210-400 nm
**Injection Volume**	1.0 uL
**BPR**	125 BarG
**Isocratic Conditions**	30:70 MeOH:CO2 (0.2% v/v NH3)

8.3 qNMR method

Sample Preparation

Around 2 mg of in-house compound or caffeine (Sigma reference standard, C1778) was accurately weighed (Mettler Toledo XP26 micro balance) and dissolved in 500 µL of deuterated DMSO (Euriso-top, D034T). 2 samples were prepared for each compound. 160 uL of the solution was transferred to 3 mm NMR tube (Bruker Z112272), 3 tubes were prepared from the same sample.

NMR experiment

NMR data was collected on a Bruker Avance NEO spectrometer equipped with 600 MHz magnet and 5 mm TCI Cryoprobe. The ^{1}H spectrum was referenced to the internal deuterated solvent. The operating frequency for ^{1}H was 600 MHz. All NMR data were acquired at the temperature of 298 K. All data were acquired and processed using Bruker Topspin 4.0. The quantitative 1H-NMR spectrum (@qHNMR3) was

acquired using a Bruker standard 1D zg pulse sequence with 32 scans. The sweep width was 19.8 ppm, and the FID contained 64k time-domain data points. Relaxation delay was set to 20 sec. The average value of the integrals in the aromatic region (7-9 ppm) was used to obtain the absolute concentration of the compound. Quantitative Data was analysed using Topspin 4.0.5, Eretic function. For each sample, three NMR tubes were prepared as described above, and the averaged value was used to obtain the purity value for each sample. The average purity of the two samples was quoted as the final compound purity. The purity of caffeine control samples (98.3%) was consistent with its specification (98.5-101.0%). The SD between three NMR tubes was less than 3% and the SD between two samples was 0.2%.

8.4 qNMR analysis for CCT373566

The purity analysis of **CCT373566** was found to be 91.5% (SD 3.4%) (Table S5).

Example ^{1}H NMR spectrum used in CCT373566 qNMR analysis

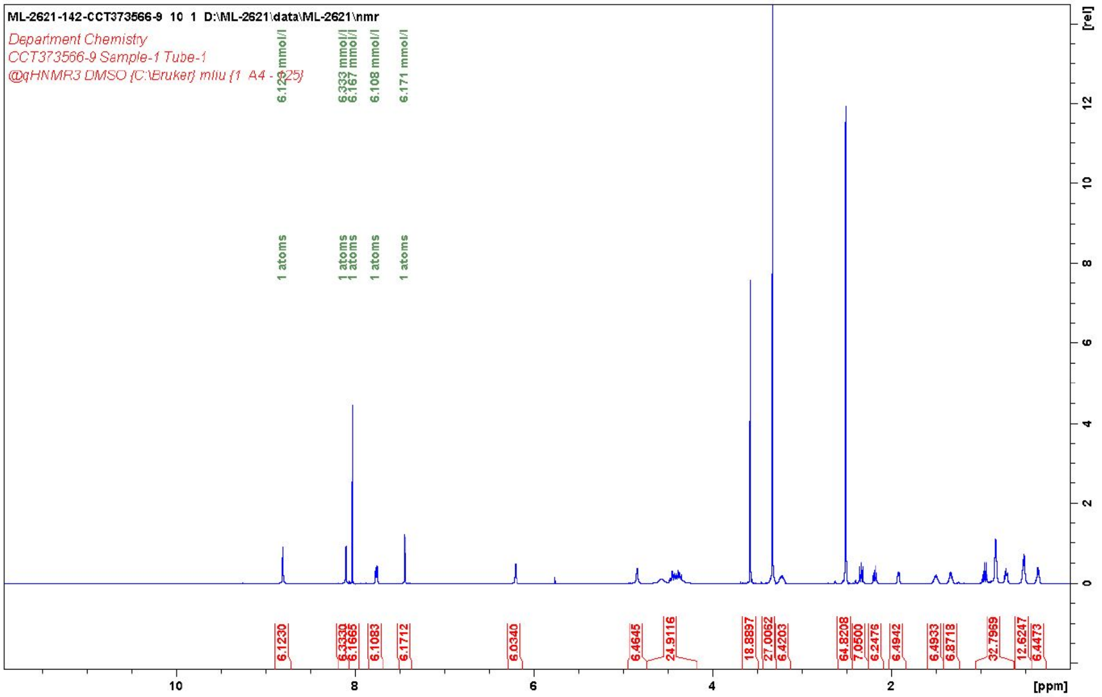

9. Supplementary Tables

Supplementary Table S1:

TR-FRET summary statistics and individual replicate values

No.	BCL6 TR-FRET IC_{50} Geometric Mean (nM)	BCL6 TR-FRET IC_{50} individual replicates (nM)	BCL6 TR-FRET pIC_{50} mean	BCL6 TR-FRET pIC_{50} SD	BCL6 TR-FRET pIC_{50} SEM	n
**2 CCT369260**	522.7	734.8, 462.1, 671.4, 736.2, 1537.3, 553.2, 235.6, 980.3, 188.7, 306.1, 417.6	6.2818	0.2706	0.0816	11
**3**	237.6	226.8, 247.3, 239.3	6.6241	0.019	0.0110	3
**4**	86.1	93.8, 54.6, 100.4, 106.7	7.0652	0.1338	0.0669	4
**5**	95.1	101.1, 99.6, 94.3, 86.2	7.0218	0.0314	0.0157	4
**8a**	3.5	3.2, 3.3, 4.0	8.4571	0.0549	0.0317	3
**8b**	4.6	4.8, 4.7, 4.4	8.3342	0.0177	0.0102	3
**9a**	1.5	1.3, 2.2, 2.2, 0.9, 4.0	8.8118	0.1948	0.0974	4
**9b**	4.1	3.4, 3.8, 5.0, 4.2	8.3907	0.0667	0.0334	4
**10a**	3.6	3.4, 4.4, 3.2	8.4394	0.0764	0.0441	3
**10b**	2.9	3.0, 3.5, 2.6, 2.5	8.5388	0.0655	0.0328	4
**11a**	3.3	3.9. 3.2, 3.0, 3.3	8.4757	0.0475	0.0238	4
**11b**	3.6	3.3, 3.0, 4.8	8.4399	0.1096	0.0633	3
**12a CCT373566**	2.2	2.9, 1.8, 2.3, 1.8, 1.8, 1.2, 2.7, 2.3, 3.8, 1.9, 2.2, 1.7, 2.6, 2.6, 2.1, 2.1, 2.6, 2.1, 2.3, 2.4	8.6638	0.1066	0.0238	20
**12b CCT373567**	2.8	3.9, 2.4, 2.4, 2.6, 2.8	8.5554	0.0853	0.0381	5
**13a**	9.9	11.4, 9.9, 8.6	8.0053	0.0608	0.0351	3
**13b**	6.0	4.9, 6.4, 6.8	8.2236	0.0784	0.0453	3
**14a**	2.5	1.7, 2.4, 3.0, 3.1	8.6087	0.1168	0.0584	4
**14b**	3.0	2.6, 3.1, 3.2, 2.9, 3.1	8.5254	0.0374	0.0167	5
**15a**	36.5	48, 29.5, 34.4	7.4377	0.1082	0.0625	3
**15b**	24.6	23.8, 27.6, 22.6	7.6089	0.0451	0.0260	3

Supplementary Table S2:

MSD summary statistics and individual replicate values

No.	MSD degrader assay [OCI-LY1] DC_{50} Geometric Mean (nM)	MSD degrader assay [OCI-LY1] DC_{50} individual replicates (nM)	MSD degrader assay [OCI-LY1] pDC50 mean	MSD degrader assay [OCI-LY1] pDC50 SD	MSD degrader assay [OCI-LY1] DC_{50} SEM	MSD degrader assay [OCI-LY1] D_{max} mean (%)	MSD degrader assay [OCI-LY1] D_{max} individual replicates (%)	n
**2 CCT369260**	54.4	46.8, 66.7, 54.4, 47.2, 56.6, 76.9, 69.2, 57.8, 73.7, 43.5, 65.8, 28.6, 37.5, 54.1, 60.8, 52.4, 51, 51, 36.3, 34.8, 70, 48.9, 26.7, 54.2, 41.2, 40.5, 49.7, 45.3, 47.5, 63.5, 37, 62, 29.3, 41.3, 74.7, 96.3, 40.6, 51.2, 57.8, 93.1, 88.5, 61, 212.5, 110.9, 25.5, 43, 303.1, 40, 220.6, 100.4, 44.4, 48.6, 68.7, 82.8, 41.4, 36.2, 25.8, 47.9, 26.7, 45.7, 32.8	7.2642	0.211	0.02702	99.29	99.8, 99.8, 97.7, 99.1, 97.9, 98.4, 97.1, 96.9, 101.0, 101.4, 101.4, 92.7, 98.6, 101.2, 102.7, 86.1, 99.5, 101.5, 98.6, 98.1, 95.9, 98.3, 98.2, 97.1, 99.8, 97.2, 103.6, 95.3, 100.4, 89.5, 101.2, 98.7, 98.2, 102.6, 102.6, 100.6, 100.9, 97.3, 98.8, 99.0, 108.5, 100.7, 104.6, 101.1, 101.6, 104.6, 99.8, 100.7, 101.4, 99.1, 98.7, 99.5, 101.1, 99.4, 98.1, 99.2, 97.5, 99.2, 98.7, 99.7	61
**3**	275.9	245.5, 310	6.5593	0.0716	0.0506	77.55	81.0, 74.1	2
**4**	80.2	93.2, 70.2, 78.9	7.0957	0.0619	0.0357	87.60	86.2, 84.7, 91.9	3
**5**	34.5	66, 18	7.4626	0.3992	0.2823	88.84	91.035, 86.653	2
**8a**	7.1	4.3, 11.6, 7.1	8.1499	0.2149	0.1241	59.57	60.5, 50.7, 67.5	3
**8b**	>2000	>2000	-	-	-	<30	<30	1
**9a**	2.7	2.5, 3.0	8.5622	0.0474	0.0335	82.83	76.7, 89.0	2
**9b**	>2000	All >2000	-	-	-	44.73	56.1, 58.2, 19.9	3
**10a**	2.2	1.7, 2.8	8.6565	0.1498	0.1059	67.00	72.367, 61.63	2
**10b**	>2000	>2000	-	-	-	<30	<30	1
**11a**	9.8	8.3, 8.9, 12.7	8.0099	0.0984	0.0568	56.40	53.6, 60.7, 54.9	3
**11b**	>2000	>2000	-	-	-	<30	<30	1
**12a CCT373566**	0.7	0.9, 0.9, 0.9, 0.4, 0.6, 1.3, 1, 0.9, 1.5, 0.5, 0.5, 0.8, 1.2, 0.3, 0.6, 0.6, 0.4	9.1465	0.2106	0.0511	91.52	89.8, 90.3, 91.7, 95.3, 95.3, 94.5, 93.1, 94.4, 94.1, 92.7, 96.2, 84.1, 84.0, 89.6, 88.7, 89.5, 92.4	17
**12b CCT373567**	>2000	All >2000	-	-	-	<30	All <30	5
**13a**	>2000	>2000	-	-	-	<30	<30	1
**13b**	>2000	>2000	-	-	-	<30	<30	1
**14a**	>2000	All >2000	-	-	-	34.93	35.0, 34.9	2
**14b**	>2000	All >2000	-	-	-	<30	<30	2
**15a**	6.3	16, 1.7, 9.3	8.1987	0.5074	0.2929	85.55	88.4, 83.3, 85.0	3
**15b**	32.6	19.4, 39.6, 44.8	7.4874	0.1956	0.1129	72.07	73.4, 70.6, 72.2	3

Supplementary Table S3:

Crystallographic data collection and refinement statistics.

Crystal system	Flag-BCL6
Ligand	**CCT373566**
PDB code	7QK0
Crystal	
Space group	P 6_1 2 2
Unit cell dimensions (a/b/c in Å)	66.84/66.84/167.28
Unit cell angles (α/β/γ in °)	90/90/120
Data collection and processing	
Beamline	DLS I03
Wavelength (Å)	0.9763
Integration program	XDS
Reduction program	AIMLESS
Resolution range	41.82 – 1.96
Number of unique reflections [a]	16722 (1137)
Completeness [a]	100 (100)
Redundancy [a]	6.8 (6.5)
R_{merge} (%) [a]	11.9 (206.8)
I/σ(I) [a]	8.0 (1.0)
$CC_{1/2}$ [a, b]	0.997 (0.359)
Refinement	
Program	BUSTER
R_{work} (%)	20.11
R_{free} (%)	21.98
Number of residues	131
Number of water molecules	103
Average B-factor (Å^2)	42.91
Ramachandran favoured (%)	98.45
Ramachandran outliers (%)	0
RMSD bonds (Å)	0.012
RMSD angles (°)	1.564

[a] Values in parentheses are for the highest resolution shell.

[b] Half-dataset correlation coefficient, see: Karplus, P. A.; Diederichs, K. Linking crystallographic model and data quality. *Science* **2012**, *336*, 1030−1033.

Supplementary Table S4:

*Composition of solution formulation for **CCT373566** and **CCT369260***

Component	Amount (%)	Amount per 10 mL (5 mg/mL)
**CCT369260 (2)/CCT373566 (12a)**		50 mg
**DMSO**	10% v/v	1 mL
**Kolliphor HS15**	20% v/v	2 mL
**PEG400**	30% v/v	3 mL
**HPMC 1.25% aq**	40% v/v	4 mL

Supplementary Table S5:

*qNMR purity analysis for **CCT373566***

Compound	Batch	Sample	tube	Weight (mg)	MW	Volume (uL)	Calc Conc. (mM)	qNMR Conc. (mM)	Purity (%)	Average	RSD	Average2	SD (n=2)
CCT373566	9	1	1	1.942	547.00	500	7.101	6.181	87.0%	88.1%	1.0%	**91.5%**	**3.4%**
CCT373566	9	1	2	1.942	547.00	500	7.101	6.25	88.0%				
CCT373566	9	1	3	1.942	547.00	500	7.101	6.346	89.4%				
CCT373566	9	2	1	3.25	547.00	500	11.883	11.279	94.9%	94.9%	0.6%		
CCT373566	9	2	2	3.25	547.00	500	11.883	11.356	95.6%				
CCT373566	9	2	3	3.25	547.00	500	11.883	11.193	94.2%				

10. <u>Supplementary Figures</u>

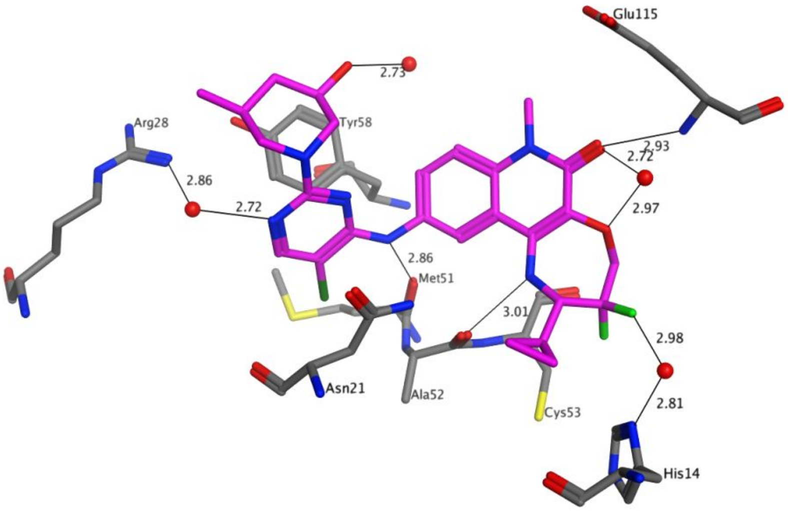

Supplementary Figure S1:

X-ray structure of the BCL6 BTB domain with bound ligand ***CCT373566*** *(PDB: 7QK0, magenta) highlighting key distances between protein and ligand.*

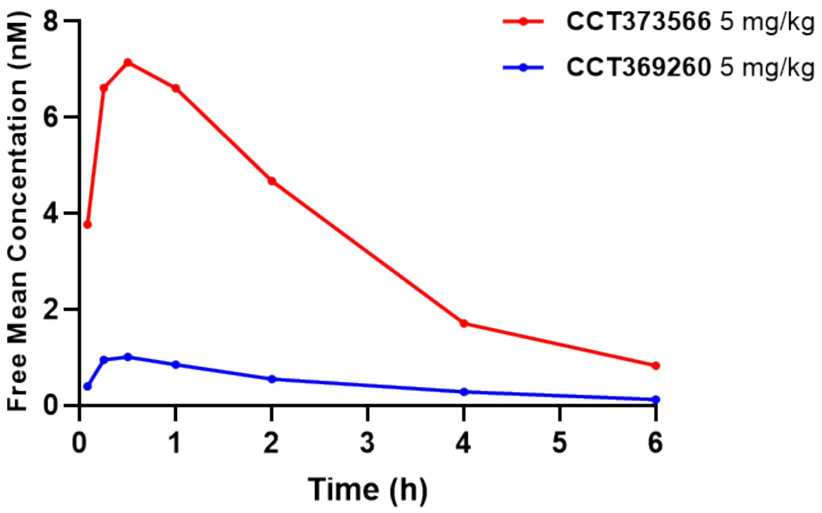

Supplementary Figure S2:

Free mean mouse (BALB/c) blood concentrations (nM) of ***CCT373566*** *(red) and* ***CCT369260*** *(blue) after po dosing at 5 mg/kg.*

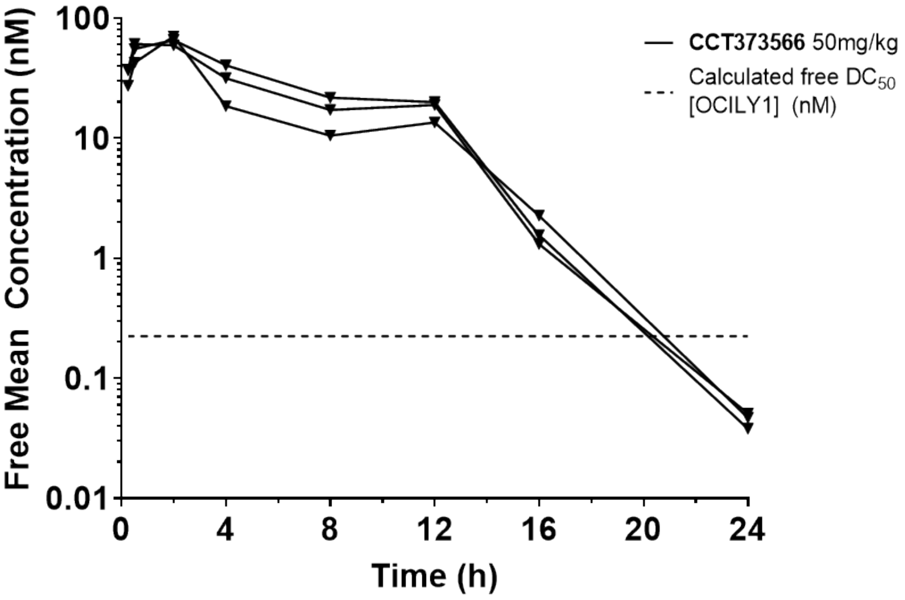

Supplementary Figure S3:

*Free mouse (SCID) blood concentrations of **CCT373566** after po dosing at 50 mg/kg (black triangles, solid line) vs the free DC_{50} value (as calculated from from the free fraction as measured in the OCI-Ly1 DC_{50} assay medium).*

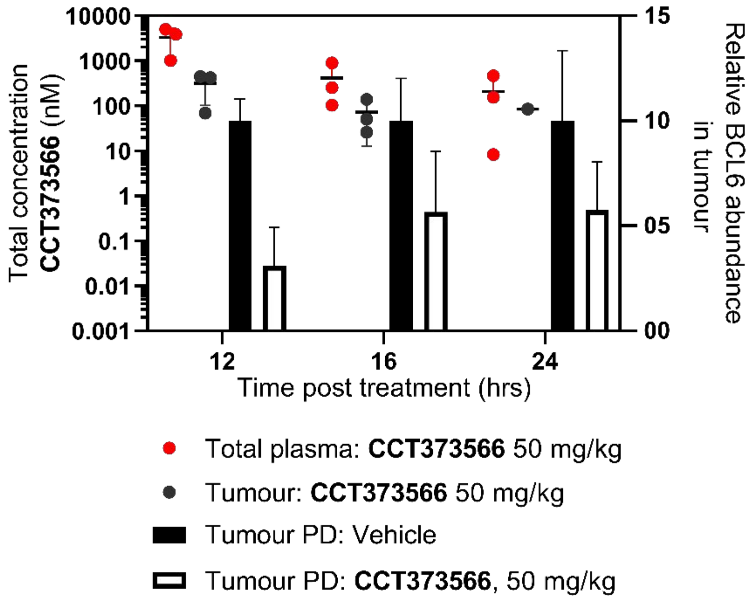

Supplementary Figure S4:

*PK/PD study with **CCT373566** at 50 mg/kg po. Tumour xenografts were prepared by subcutaneous injection of 1.5 × 10^7 OCI-Ly1 cells in female SCID mice, with dosing of compound commencing 20 days after injection, to mice with xenografts between 0.5 and 0.8 cm^3, as described in more detail in **7.3.2**. Sampling took place at 12, 16 and 24 h postdose. All experiments were carried out according to the U.K. guidelines for animal experimentation. BCL6 levels in tumor were quantified using capillary electrophoresis and normalized to a GAPDH loading control and are shown as black (vehicle-treated) or white (compound treated) bars. Total compound levels at 12–24 h are shown in the plasma (red dots) and tumour (black dots).*

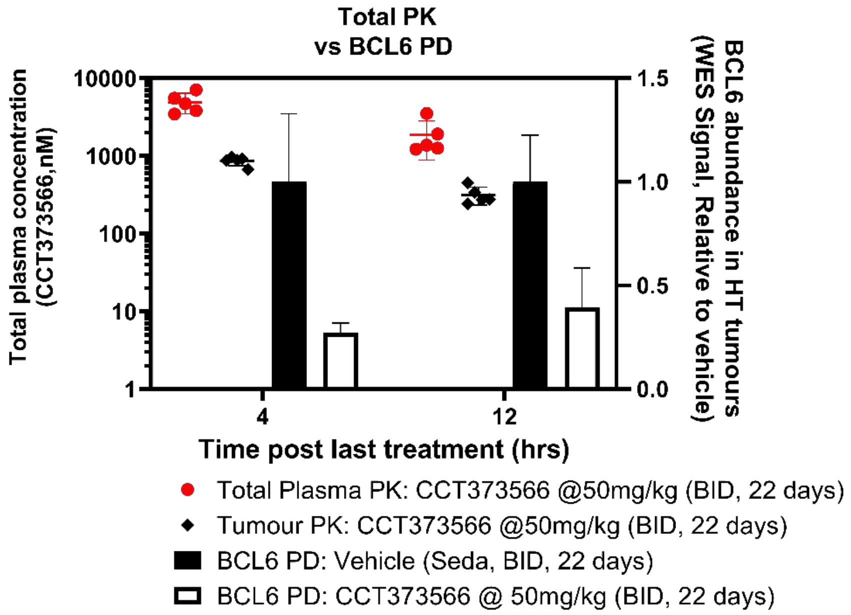

Supplementary Figure S5:

*PK/PD study with **CCT373566** at 50 mg/kg po BID for 22 days. Tumour xenografts were prepared by subcutaneous injection of 1 × 10^7 HT cells in female SCID mice, with dosing of compound BID commencing 20 days after injection, to mice with xenografts between 0.5 and 0.8 cm^3 and lasting 22 days, as described in more detail in **7.3.4**. Sampling took place at 4 and 12 h post final dose. All experiments were carried out according to the U.K. guidelines for animal experimentation. BCL6 levels in tumor were quantified using capillary electrophoresis and normalized to a GAPDH loading control and are shown as black (vehicle-treated) or white (compound treated) bars. Total compound levels at 4 and 12 h are shown in the plasma (red dots) and tumour (black dots).*

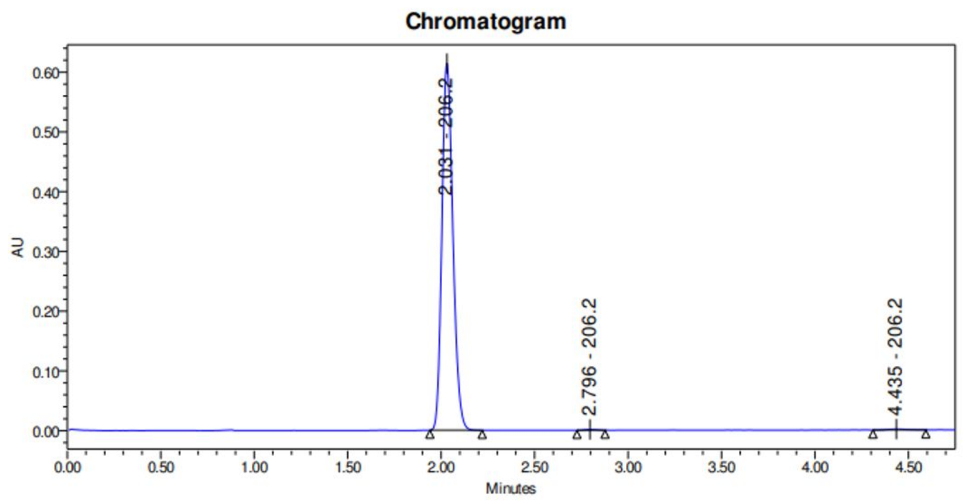

Peak Results

	Retention Time (min)	Area (µV*sec)	% Area	Width @ 50%
1	2.03	2501508	99.3	0.06307
2	2.80	5964	0.2	0.07273
3	4.44	10817	0.4	0.10996

**Column Details**	Amy-C (4.6mm x 250mm, 5um)
**Column Temperature**	40°C
**Flow Rate**	4 mL/min
**Detector Wavelength**	210-400 nm
**Injection Volume**	1.0 uL
**BPR**	125 BarG
**Isocratic Conditions**	10:90 MeOH:CO2 (0.2% v/v NH3)

Supplementary Figure S6:

Chiral HPLC analysis of intermediate ***22a***

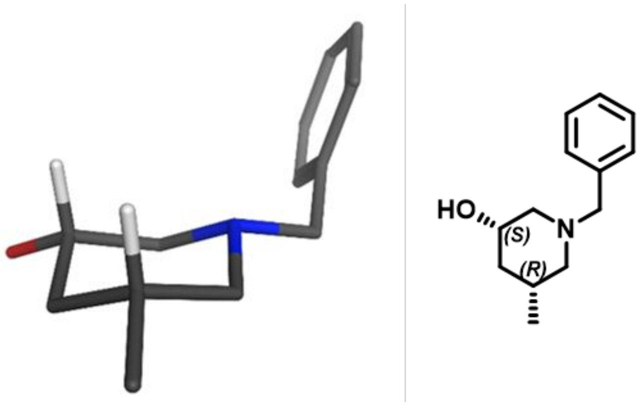

Supplementary Figure S7:

X-ray crystal structure of intermediate ***22b****.*

11. References for supplementary information

(1) Kabsch, W. XDS. *Acta Crystallographica Section D* **2010**, *66* (2), 125-132.

(2) Evans, P. Scaling and assessment of data quality. *Acta Crystallographica Section D* **2006**, *62* (1), 72-82.

(3) McCoy, A. J.; Grosse-Kunstleve, R. W.; Adams, P. D.; Winn, M. D.; Storoni, L. C.; Read, R. J. Phaser crystallographic software. *Journal of Applied Crystallography* **2007**, *40* (4), 658-674. DOI: doi:10.1107/S0021889807021206. Winn, M. D.; Ballard, C. C.; Cowtan, K. D.; Dodson, E. J.; Emsley, P.; Evans, P. R.; Keegan, R. M.; Krissinel, E. B.; Leslie, A. G. W.; McCoy, A.; et al. Overview of the CCP4 suite and current developments. *Acta Crystallographica Section D* **2011**, *67* (4), 235-242.

(4) Ghetu, A. F.; Corcoran, C. M.; Cerchietti, L.; Bardwell, V. J.; Melnick, A.; Privé, G. G. Structure of a BCOR Corepressor Peptide in Complex with the BCL6 BTB Domain Dimer. *Molecular Cell* **2008**, *29* (3), 384-391.

(5) Emsley, P.; Cowtan, K. Coot: model-building tools for molecular graphics. *Acta Crystallographica Section D* **2004**, *60* (12 Part 1), 2126-2132.

(6) G. Bricogne, E. B., M. Brandl, C. Flensburg, P. Keller, W. Paciorek, P. Roversi,; A. Sharff, O. S. S., C. Vonrhein, T.O. Womack. *BUSTER, Version 2.10.2, Global Phasing Ltd., Cambridge, United Kingdom* **2015**.

(7) O.S. Smart, T. O. W., A. Sharff, C. Flensburg, P. Keller, W. Paciorek,; C. Vonrhein, G. B. *Grade, Version 1.2.9, Global Phasing Ltd., Cambridge, United Kingdom, 2014.*

(8) Bruno, I. J.; Cole, J. C.; Lommerse, J. P. M.; Rowland, R. S.; Taylor, R.; Verdonk, M. L. IsoStar: A library of information about nonbonded interactions. *Journal of Computer-Aided Molecular Design* **1997**, *11* (6), 525-537, journal article.

(9) Chen, V. B.; Arendall, W. B., III; Headd, J. J.; Keedy, D. A.; Immormino, R. M.; Kapral, G. J.; Murray, L. W.; Richardson, J. S.; Richardson, D. C. MolProbity: all-atom structure validation for macromolecular crystallography. *Acta Crystallographica Section D* **2010**, *66* (1), 12-21. Davis, I. W.; Leaver-Fay, A.; Chen, V. B.; Block, J. N.; Kapral, G. J.; Wang, X.; Murray, L. W.; Arendall, W. B., III; Snoeyink, J.; Richardson, J. S.; et al. MolProbity: all-atom contacts and structure validation for proteins and nucleic acids. *Nucleic Acids Research* **2007**, *35* (suppl_2), W375-W383.

(10) Coles, S. J.; Gale, P. A. Changing and challenging times for service crystallography. *Chem. Sci.* **2012**, *3* (3), 683-689.

(11) Wider, G.; Dreier, L. Measuring Protein Concentrations by NMR Spectroscopy. *Journal of the American Chemical Society* **2006**, *128* (8), 2571-2576. Lin, M.; Tesconi, M.; Tischler, M. Use of 1H NMR to facilitate solubility measurement for drug discovery compounds. *International Journal of Pharmaceutics* **2009**, *369* (1), 47-52. DOI: Saal, C.; Petereit, A. C. Optimizing solubility: Kinetic versus thermodynamic solubility temptations and risks. *European Journal of Pharmaceutical Sciences* **2012**, *47* (3), 589-595.

(12) Workman, P.; Aboagye, E. O.; Balkwill, F.; Balmain, A.; Bruder, G.; Chaplin, D. J.; Double, J. A.; Everitt, J.; Farningham, D. A. H.; Glennie, M. J.; et al. Guidelines for the welfare and use of animals in cancer research. *British Journal of Cancer* **2010**, *102* (11), 1555-1577.
